# Equine Responses to Acceleration and Deceleration Cues May Reflect Their Exposure to Multiple Riders

**DOI:** 10.3390/ani11010066

**Published:** 2020-12-31

**Authors:** Jessica McKenzie, Kate Fenner, Michelle Hyde, Ashley Anzulewicz, Bibiana Burattini, Nicole Romness, Bethany Wilson, Paul McGreevy

**Affiliations:** 1 School of Life and Environmental Sciences, Faculty of Science, University of Sydney, Sydney, NSW 2006, Australia; 2 Sydney School of Veterinary Science, Faculty of Science, University of Sydney, Sydney, NSW 2006, Australia; kate@kandooequine.com.au (K.F.); michellehyde1@bigpond.com (M.H.); aanz4186@uni.sydney.edu.au (A.A.); bbur2426@uni.sydney.edu.au (B.B.); nrom3994@uni.sydney.edu.au (N.R.); Bethany.wilson@sydney.edu.au (B.W.); paul.mcgreevy@sydney.edu.au (P.M.)

**Keywords:** behaviour, equitation science, negative reinforcement, operant conditioning, rider skill, welfare

## Abstract

**Simple Summary:**

Successful horse training depends on riders giving clear and consistent cues. When cues are inconsistent, the horse may become confused, frustrated, or unresponsive. It is likely that each rider or horse trainer differs in the way they deliver training cues because humans vary in their weight, height, riding style, handedness, experience, and skill level. This study explored relationships between the number of people to ride or handle a horse and the horse’s response to training cues. Data describing 1819 horses and ponies were obtained from the Equine Behavior Assessment and Research Questionnaire (E-BARQ), an online global survey of horse owners and caregivers. The number of riders or handlers showed a significant relationship with two behavioural indices. Specifically, as the number of riders or handlers increased, horses were more difficult to accelerate and less difficult to decelerate compared to horses with fewer riders or handlers. This could indicate that an increase in rider or handler numbers is associated with those horses becoming relatively more unresponsive to leg and whip cues than to rein cues.

**Abstract:**

It is logical to assume that horses with multiple riders encounter variation in application of training cues. When training cues are inconsistent, we expect to see a decrease in trained responses or an increase in conflict behaviours. This study investigated the relationship between the number of people that regularly ride or handle a horse and the horse’s response to operant cues. Data on 1819 equids were obtained from the Equine Behavior Assessment and Research Questionnaire (E-BARQ), an online global survey of horse owners and caregivers. Three mutually independent indices (acceleration, deceleration, and responsiveness) were derived from a parallel analysis of E-BARQ items related to acceleration and deceleration cues. These indices were then subjected to multivariable modelling against a range of dependent variables including horse and human demographics, horse management, and the number of riders or handlers. The number of riders or handlers was a significant predictor for two out of three indices. As the number of riders or handlers increased, horses were more difficult to accelerate (regression coefficient = 0.0148 ± 0.0071; *p* = 0.0366) and less difficult to decelerate (regression coefficient = −0.017 ± 0.008; *p* = 0.030) than those with fewer riders or handlers. These findings suggest that horses’ responses to rein tension cues are more persistent than their responses to leg pressure or whip cues. Alternatively, horses with these responses may be actively selected for multiple rider roles. Longitudinal studies of this sort should reveal how the number of riders or handlers affects horse behaviour and could lead to safer and more humane equestrian practices.

## 1. Introduction

Since the mid-20th century there have been substantial advances in our understanding of animal cognition and learning [1]. However, these advances have been slow to gain traction in equestrian communities that often value traditions and esoteric knowledge over science [2]. A lack of understanding of the equine learning process can lead to training or management practices that confuse horses [3] and can result in training deficits or the emergence of undesired behaviours [4]. There is substantial evidence to highlight the importance of clear and consistent training cues for the mental wellbeing of captive and domestic animals [5,6].

The field of equitation science seeks to educate horse owners and caregivers about effective and humane methods of training and horse management [6]. Equitation science training principles are informed by learning theory and take into consideration the ethology and cognitive ability of the horse [6]. Most horse training techniques rely on a form of associative learning known as operant conditioning. This is the process by which an animal learns to associate its actions with a reinforcing or punishing outcome.

Learning theory is underpinned by the concept of four operant conditioning quadrants, namely positive reinforcement, negative reinforcement, positive punishment, and negative punishment. Reinforcement makes behaviours more likely to occur in the future and punishment makes behaviours less likely to occur in the future. Negative reinforcement is ubiquitous in horse riding since, once a rider is sitting on a horse, pressure cues are effectively unavoidable, and so their removal can be used to reinforce desirable locomotory responses, such as acceleration. Some horse training uses positive and negative reinforcement together, an approach known as combined (or blended) reinforcement [1].

Combined reinforcement can be highly effective when correctly timed. Of course, negative reinforcement always needs to be correctly timed to avoid becoming punishment. When the release of pressure signals is not timed correctly, the horse may be subjected to pressures that are excessive or unrelenting. Any delay in the removal of a pressure cue results in positive punishment for the horse; thus, making the behaviour less likely to occur in the future [7]. Excessive pressures can also trigger the horse’s natural antipredator response [8] that includes potentially dangerous behaviours such as bucking, rearing and bolting and is usually associated with a heightened state of fear or arousal [8]. Conversely, if pressure cues are insufficient to motivate the horse, it may learn to ignore the cues through the process of habituation [1]. This is a natural adaptive mechanism that allows animals to become progressively less responsive to any biologically insignificant stimulus that is regularly encountered [1]. Operant conditioning could inadvertently serve to reinforce these behaviours if the horse succeeds in escaping from pressure, for example if the rider is thrown from the saddle [8].

When horses are ridden by multiple riders, any variation in riders’ morphometrics, handedness, riding style, or skill level could expose the horse to inconsistent application of operant cues, notably pressure cues via the riders’ legs and hands. If training signals and reinforcements are applied inconsistently, we would expect to see a reduction of responsiveness or an increase in escape and avoidance behaviours [4,7,8]. Either of these outcomes could lead to an increased risk of injury to the rider as well as exposing the horse to negative welfare outcomes.

The horse’s exposure to multiple riders has been linked to extreme conflict behaviours such as bucking, rearing, and bolting [4]. In a survey of British leisure riders (*n* = 1326), Hockenhull and Creighton found horses with three regular riders were at a higher risk of displaying extreme conflict behaviour than horses with only one rider [4]. This could indicate that horses with three riders encounter more stress and confusion in their work than horses with one rider. However, it is unclear whether the conflict behaviours emerged in response to multiple riders. Other explanations merit consideration. For example, it is possible that additional (relatively skilled) riders were recruited to help the (relatively unskilled) leisure rider address a training problem that was beyond their skill level.

Horse riding is a dangerous activity with a risk of severe injury or death [9]. The mortality rate of horse riders is reported to be higher than any other sport [10,11]. Injuries to participants are both more frequent and more severe than those incurred in other recreational activities [10,11,12]. Horse behaviour is frequently reported to be the most significant risk factor in horse-related injuries [11,13]. Therefore, a better understanding of factors that affect horse behaviour could help to reduce the risk to human participants in the sport.

Knowledge of horse behaviour can also be used to improve horse welfare. Undesirable behaviour in horses may emerge as a response to aversive experiences, such as pain, fear, or confusion [8]. Such behaviours can also compromise the welfare of horses when trainers rely on punishment based methods, suboptimal negative reinforcement or use of aversive equipment [3,7]. Such methods and equipment have the potential to compromise horse welfare [3] and could also cause an escalation of potentially dangerous behaviour if the horse’s fight-or-flight response is triggered [14]. Undesirable behaviour can also diminish the perceived value of the horse, causing the horse to be sold, auctioned, or euthanased [15]. Apart from horse welfare and rider safety, the issues of wastage, loss of performance potential, and the impact of variations in initial training of the horse also merit consideration when one explores the influences on and the impact of equine behaviour.

There are many situations in which horses may be exposed to multiple riders or handlers. Privately owned horses may be primarily ridden or handled by their owners but also by coaches, trainers, friends and family. Horses kept in professional training stables or agistment or livery centres would commonly be handled and sometimes ridden by grooms and other staff. Routine husbandry procedures, such as farriery and dentistry, may also require the horse to be handled by unfamiliar humans.

Horses in riding school or trail riding establishments are typically exposed to several riders, often including novice riders. Novice riders differ from experienced riders in their posture, synchronicity and balance [16,17,18,19]. These differences could inhibit the novice rider’s ability to deliver clear signals and a timely release or reward. However, studies have reported no effect of rider experience level on ridden horse behaviour [16] or biological markers of stress [20,21,22]. Each of these studies had limitations such as small sample sizes [16,20,21,22], inadequate controls [20] and limited scope [21,22]. Due to these limitations, the question of how novice riders affect their mounts requires further investigation.

Equine ridden behaviour is a complex outcome with many contributing factors. The horse’s behaviour under saddle is thought to be influenced by intrinsic factors, such as horse breed, sex, and age. While one recent study found that common equestrian preconceptions about the behaviour of mares were largely unfounded [23], a horse’s breed can influence its behaviour, temperament, and learning ability [24,25]. The behaviour of horses with multiple riders may also reflect common management flaws such as poorly fitting saddles and bridles [4,26], inappropriate diet [27], or untreated musculoskeletal pain [28]. These are potentially confounding issues because pain and stress-related behaviours are not easily distinguished from behaviours arising from errors in training [28,29].

The Equine Behavior Assessment and Research Questionnaire (E-BARQ) was developed to investigate how horse management and training interact with horse behaviour. This international, online survey of horse owners and caregivers has the potential to address a series of knowledge gaps in current global horse management and training practices. The current study uses E-BARQ data to investigate the relationship between number of riders or handlers and the horse’s response to operant cues. We predicted a decrease in responsiveness to operant cues with increase in number of riders. These cues include the use of leg or whip to accelerate and the use of the rein tension cues to decelerate. They were selected because they represent fundamental aspects of ridden horse training [6].

## 2. Materials and Methods

### 2.1. Survey Design and Distribution

E-BARQ is an ongoing project with ethics approval from the University of Sydney Human Research Ethics Committee (approval number: 2012/656). The pilot questionnaire was developed in consultation with an international panel of nine equine professionals [30] and subjected to a Rotated Principal Component Analysis, resulting in the current survey of 97 validated matrix-style questions [31]. It includes 42 demographic items concerning the respondent and the focal horse. The survey then branches into ridden and non-ridden sections, which contain 268 and 218 items, respectively. These sections cover a wide range of management, training, and behaviour questions. A copy of the survey is presented as Supplementary Material.

The survey was built using Qualtrics [32] and is available online at www.e-barq.com, the data for this study were collected between November 2019 and July 2020. For the purpose of this and concurrent studies, the survey link was distributed to the email lists of Equitation Science International (https://www.esi-education.com), Kandoo Equine (https://www.kandooequine.com) and *Horses and People* magazine (https://horsesandpeople.com.au/). The survey was also promoted via email and on social media platforms.

### 2.2. Selection and Construction of Dependent Indices

The E-BARQ questionnaire was searched for items describing behavioural responses to deceleration and acceleration cues. Twenty-two candidate items were identified and subjected to a Rotated Principal Component Analysis using varimax rotation. Principal Component Analysis is a method to extract latent, not directly measured variables, from a large and complex dataset. Rotation makes the components easier to interpret [30].

The Psych package [33] of R statistical software [34] was used to compare the scree of components of the standardised observed data with that of a random data matrix of the same size. A parallel analysis was used to determine the number of components (RC) to extract and rotate. This analysis revealed four relatively uncorrelated RCs. The first three RCs were selected to construct behavioural indices: Acceleration, Deceleration, and Responsiveness. The fourth RC (Travelling) was dropped because it was deemed less relevant to the current research question. Items were considered for an index if they had a loading of 0.4 or higher, unless they loaded more strongly to another component. Items were removed from an index if removal improved the value of Cronbach’s alpha.

The selected E-BARQ items were constructed using Likert scales with five levels and provided a sixth option of “not observed/applicable”. In most cases, numerical values were assigned to the Likert scales as follows: Never = 1, Rarely = 2, Sometimes = 3, Usually = 4, Always = 5; or Strongly disagree = 1, Disagree = 2, Neutral = 3, Agree = 4, Strongly agree = 5. However, if an item was negatively correlated with other items in the index, the values were reversed to ensure meaningful scoring.

The score for each behavioural index (Acceleration, Deceleration, or Responsiveness) was the sum of scores for each item that contributed to the index. To account for missing values, available scores were summed, divided by the number of scores available and multiplied by the number of items in the index. This ensured the missing value was weighted according to similar items, rather than imputing an overall mean.

### 2.3. Independent Variable Selection

The number of riders was derived from the E-BARQ question: “Using the past 12 months as a guide, how many different handlers/riders are likely to ride or handle [horse name] per month?”. Responses were assigned a numerical value from 1 to 6 as follows: “I am the only person to ride or handle this horse” = 1; “2 people” = 2; “3 people” = 3; “4 people” = 4; “5 people” = 5; “6–10 people” or “11 or more people” = 6.

Basic horse and human demographic variables were forced into the model. Other independent variables of interest were assessed for inclusion in the final model by univariable analysis of the dependent indices. Those variables with *p* < 0.2 were selected for inclusion.

### 2.4. Multivariable Modelling

The effect of independent variables on each of the indices was then assessed by multivariable modelling. The full model contained the forced variables and other variables selected from univariable analysis. The least significant optional term was then removed until all optional terms were *p* < 0.2. Rider gender by age interactions, horse sex by horse age interactions and horse age by breed interactions were added individually to the final model and retained if *p* < 0.2.

## 3. Results

### 3.1. Sample Size and Demographics

The study population was a self-selected convenience sample of horse owners and caregivers. At the time of the current study, 1322 respondents had completed the E-BARQ survey for 1819 horses. The respondents were predominantly female (*n* = 1258), with only 48 male respondents, seven gender nonconforming and nine undisclosed. Respondents were residents of 32 countries, with Australia (*n* = 562) and the United States of America (*n* = 214) being the most highly represented (Table 1). Most age groups were well represented in the survey population (Table 2). Respondents were asked to nominate their skill level from a list of six options. Most considered themselves to be an “intermediate rider/horse handler” (*n* = 873), while 710 respondents selected “advanced rider/horse handler.” Novice (*n* = 182), beginner (*n* = 29) and elite (*n* = 22) riders/handlers were fewer in numbers, while only three respondents identified with the sixth option as “a non-rider/non-horse person”.

The horse population comprised 725 female horses (mares or fillies) and 1094 male horses, of which 1067 were castrated (geldings). The population included 1064 purebred horses and 748 crossbred horses. The purebreds represented 72 different breeds, with thoroughbreds being the most common (*n* = 319), followed by quarter horses (*n* = 137), Arabians (*n* = 56), and Standardbreds (*n* = 50). Horse age ranged from 2 months to 36 years, with an average age of 11.46 years. Horses were used for 43 different disciplines, of which dressage (*n* = 351), pleasure riding (*n* = 349), trail riding (*n* = 159), eventing (*n* = 157), and show jumping (*n* = 156) were most common.

### 3.2. Selection and Construction of Dependent Indices

#### 3.2.1. Acceleration Index

The first index was labelled Acceleration and contained seven E-BARQ items (Table 3). The Cronbach’s alpha for these seven items was 0.72 (95% CI: 0.70–0.74). The removal of no single item resulted in a higher alpha, so all seven traits were retained. As the Acceleration index contained seven traits, each with five levels, the hypothetical range of scores was 7–35. Higher scores in this index represented horses that were harder to accelerate. The index was log-transformed to correct a negative skew.

#### 3.2.2. Deceleration Index

The second index was labelled Deceleration. Eight E-BARQ items loaded strongly to this index and were therefore considered for inclusion (Table 4). Removal of item Q61_1 raised the alpha from 0.80 to 0.81 (95% CI: 0.80–0.82). Therefore, only the other seven items were used to construct this index. The Deceleration index had a hypothetical range of 7 to 35. Horses with higher scores in this index were more difficult to decelerate. The distribution of index scores was negatively skewed, corrected by log transformation.

#### 3.2.3. Responsiveness Index

The third index was named Responsiveness. There were four candidate E-BARQ items for this index (Table 5). However, the removal of item Q60_15 raised the alpha from 0.78 to 0.83 (95% CI: 0.82–0.85). Therefore, only the other three items were used to construct this index. Composed of three traits with five levels each, the hypothetical range of this index was 3–15. Unlike the direction of the other two indices, horses with a high score in Responsiveness were more responsive to rein cues. The index had a strong negative skew, which was improved by reversing the scores, taking the square root of the score and then reversing the scores again.

### 3.3. Independent Variable Selection

#### 3.3.1. Number of Riders

The primary variable of interest to this study was the number of riders or handlers a horse is regularly exposed to. Horses with one (*n* = 659) or two (*n* = 747) riders or handlers made up 77% of the responses, with relatively few horses exposed to three or more riders or handlers (Table 6).

#### 3.3.2. Univariable Modelling of Independent Variables

Univariable analysis of the dependent indices was used to select explanatory variables for the final models. Basic human demographics, horse demographics, and the number of riders were forced into all three models, regardless of *p*-value. Details of both summer and winter housing were obtained by the survey. To avoid collinearity, summer housing was selected at random using the sampling function from the base package of R. Other variables with *p* < 0.2 were selected for inclusion in the final models (Table 7).

### 3.4. Multivariable Modelling

#### 3.4.1. Acceleration Model

The final model for the Acceleration index took the form of:

Acceleration index ~ Number of riders + Gender + Country + Age + Horse sex + Horse age + Horse breed + Discipline + Respondent Experience + Summer housing + Respondent skill level + Horse sex: Age interaction

The model had a residual standard error of 0.2792 on 1377 degrees of freedom. Multiple R-squared was 0.1549 and adjusted R-squared was 0.1064. The *f*-statistic was 3.195 on 79 and 1377 degrees of freedom (*p* < 2.2 × 10^−16^). There was a small but significant relationship between the Acceleration index and the number of riders. As the number of riders increased, the horse became harder to accelerate (log estimate = 0.0148 ± 0.0071, *p* = 0.0366). Other significant predictors for hard-to-accelerate horses included the respondent’s self-evaluated skill level, experience level, age and country, as well as horse breed, discipline and horse age (Table 8).

#### 3.4.2. Deceleration Model

The final Deceleration model took the form:

Deceleration index ~ Number of riders + Gender + Country + Age + Horse sex + Horse age + Horse breed + Discipline + Saddle fit + Horse colour + Respondent experience + Summer housing + Respondent skill level

The model had a residual standard error of 0.3059 on 1361 degrees of freedom. Multiple R-squared was 0.1887 and Adjusted R-squared was 0.1368. The *f*-statistic: 3.638 on 87 and 1361 degrees of freedom, (*p* < 2.2 × 10^−16^).

There was a significant association between the number of riders and the Deceleration index. An increase in the number of riders was associated with horses that were easier to decelerate (log estimate= −0.017 ± 0.008, *p* = 0.030). Other significant predictors for hard to decelerate horses included respondent self-evaluated skill level, experience level, age, country and discipline, as well as horse age, breed, colour, and saddle fit frequency (Table 9).

#### 3.4.3. Responsiveness Model

The final model took the form:

Responsiveness index ~ Number of riders + Horse sex: Horse age + Gender + Country + Respondent Age + Horse sex + Horse age + Breed + Discipline + Respondent experience + Respondent skill level

The model had a residual standard error of 0.381 on 1369 degrees of freedom. Multiple R-squared was 0.09083 and adjusted R-squared was 0.04302. The *f*-statistic was 1.9 on 72 and 1369 degrees of freedom (*p* = 1.398 × 10^−50^).

**Figure 1:**
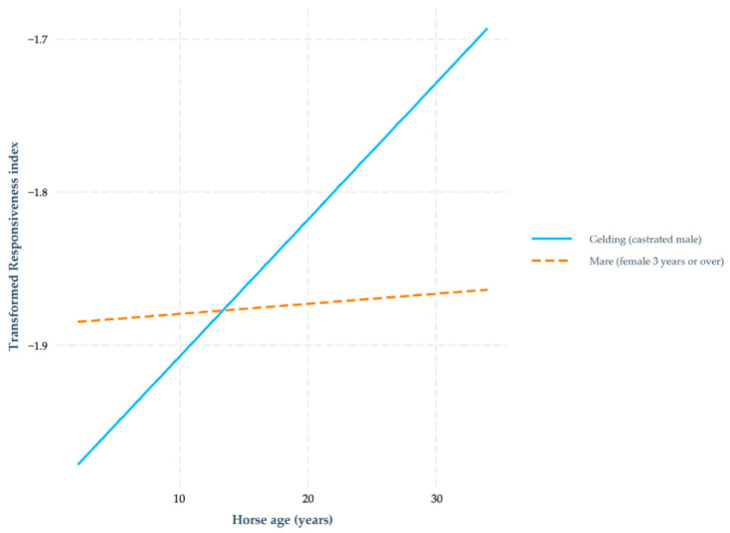
There was a significant interaction between horse age and horse sex in relation to the Responsiveness index. All horses become more responsive as they aged, but this effect was more pronounced in geldings. Younger mares were more responsive than geldings of the same age, but this effect diminished as age increased.

The number of riders was not a significant variable in this model (*p* = 0.300). Significant variables included respondent age, country, respondent experience level and self-evaluated skill level and horse breed, discipline, age and sex (Table 10). There was a significant interaction between the horse’s sex and age (Figure 1).

## 4. Discussion

The current study explored the relationship among the number of riders or handlers a horse is regularly exposed to and that horse’s behavioural indices. Significant associations were found between the number of riders and equine responses to both acceleration and deceleration cues. As the number of riders increased, horses became more difficult to accelerate and less difficult to decelerate. These results only partly agree with our prediction that an increase in riders would be associated with a decreased response to operant cues for both acceleration and deceleration. This may indicate that horses are more likely to habituate to leg and whip signals than to rein tension cues.

Habituation is an important aspect of horse training. To carry a rider, horses must become habituated to a number of persistent pressures, such as those from the girth strap around their ribcage and the weight of a rider on their back [6]. In contrast, habituation can be an undesirable result when operant cues are incorrectly applied. When using negative reinforcement, the pressure cue should be applied continuously until the horse offers the desired response. It is the removal of pressure, at the precise moment of the desired response appearing, which reinforces the desired behaviour and makes its expression more likely in future. When pressure is removed too early or too late, the rider may inadvertently punish the desired behaviour or reinforce a different behaviour [7]. When pressure is released inconsistently or not at all, the horse may learn that it is unable to make the pressure go away and become unresponsive [6].

In the current study, horses with multiple riders were more likely to be unresponsive to leg and whip cues, compared to horses with fewer riders. This infers that these horses may have been exposed to incorrect application of leg or whip cues. This could lead to a downward welfare spiral, as riders or trainers may resort to using stronger pressure or punishment techniques when a horse is seen as unresponsive. This escalation of pressure can also be achieved with equipment changes, for example by adding spurs or a whip to reinforce leg cues. The escalation of pressure could cause an already-confused horse to become withdrawn [35], or to react in explosive and unpredictable ways [4].

In all equitation science studies, it is important to avoid anthropomorphism when diagnosing the causes of unwelcome behaviour. For example, the constructs of “laziness” or “reluctance to work” imbue the horse with internal motivations that we have no evidence for. Moreover, when attributing these qualities to a horse, there is a danger that riders/trainers may overlook other possible causes of the behaviour, such as pain or confusion, and apply punitive training methods. In the current study, horses with multiple riders were less difficult to slow and stop than horses with fewer riders. On its own, this result might suggest these horses are well trained. However, when viewed in combination with the Acceleration results, this prospect becomes less likely. These horses may therefore be predisposed to move slowly and stop. This tendency could be explained by fatigue, musculoskeletal pain [28] or emotional apathy [35]. The current study did not consider how the horse’s workload or health status could affect responsiveness. Any future analysis should consider including these variables.

A horse with a good deceleration response could also be perceived as a safe horse for beginner riders [36]. Therefore, the results could indicate that horses with multiple riders, such as those used for lessons in a riding school, are selected for this quality. However, if this were the case, we would expect to see similar results for horses with beginner riders, as the same horse-rider matching considerations would apply. E-BARQ asked respondents to nominate their skill level using a six-point scale from non-rider to elite. The behavioural profile of horses with riders self-nominating as a beginner was closer to our original prediction of being less responsive to both acceleration and deceleration cues than horses with respondents self-nominating as more experienced riders. Respondents self-nominating as novice, intermediate, advanced, and elite riders were each associated with horses that were less difficult to accelerate, compared to beginner riders’ horses. Intermediate, advanced and elite riders were associated with horses that were less difficult to decelerate, compared to beginners’ horses. Intermediate, advanced and elite riders were also associated with horses that scored higher on the Responsiveness index than beginner riders. It is unlikely that a beginner rider would intentionally be matched with a horse that is unresponsive to training cues as this would be a safety concern. Therefore, it is likely that horses regularly ridden by beginner riders become less responsive to cues due to rider error. For example, the horse may become habituated to unrelenting rein pressure, or the rein signal could be overshadowed by a tightly gripping leg that applies contradictory acceleration cues. Previous studies have found novice riders to be less synchronised with their horses and have a relatively unstable seat (i.e., to move their centre of mass in ways that inadvertently send pressures cues to the horse) [16,17,18,19]. However, one study found no effect of rider skill level on the horse’s limb kinematics or ridden behaviour [16].

Horse management variables were not good predictors of ridden responses to acceleration and deceleration cues. Housing did not have a significant effect on any of the models, and frequency of saddle fit was only significant in the deceleration model. Horses that did not (yet) wear a saddle were easier to decelerate than horses wearing a saddle that was not professionally fitted. However, this was not a meaningful comparison because most horses that did not wear a saddle were unridden (*n* = 120 out of 135). These results suggest that the relationship between multiple riders or handlers and the horse’s response to training cues is independent of these management-related considerations. Further, these findings contradict previous studies that found horse behaviour may be influenced by poorly fitting equipment [4,26], pain [28] or inappropriate diet [27]. However, it was outside the scope of the current study to assess focal horses for the presence or absence of musculoskeletal pain.

Horse age was a significant variable in all three models. Horses became easier to accelerate, easier to decelerate and more responsive to rein signals as they aged. This likely reflects the effect of training and experience as the horse progresses through its ridden career. It takes time and repetition for the responses learned during operant conditioning to be reliably reproduced on cue [6]. Through classical conditioning, horses can then be trained to respond to very subtle signals, for example by responding to a shift in the rider’s weight that reliably precedes the rein cue [6]. Consideration should also be given to the possibility that poorly responding horses may have been culled.

Horse sex was significant only in the Responsiveness model and there was a significant interaction between horse age and sex. Mares were more responsive to rein cues than geldings initially, but this effect diminished with age. This could reflect the potential for mares to be used in breeding, which may cause some mares to have breaks in their training. The results could also reflect differences in the way mares and geldings are handled. If mares are perceived as more difficult to train than geldings [23], this could lead to the use of detrimental training methods. 

Several studies have established an effect of breed on temperament and behaviour [24,25]. The current findings concur in that breed was a significant term in all three models. Standardbred horses were more difficult to accelerate and less difficult to decelerate than crossbred horses. Heavy horses, Iberians, ponies, Warmbloods, and Quarter Horses were all less difficult to decelerate than crossbred horses. Heavy horses were also more responsive to rein cues than crossbred horses. Consideration should be given to the likelihood that Standardbred horses would not have been trained to respond to leg pressure at the start of their careers. This could have an effect that would not necessarily relate to temperament.

It is not clear why results show that horses from Mexico and Belgium were less difficult to accelerate or decelerate than horses from Australia, nor why horses from Belgium were more responsive to rein cues and horses from South Africa were less responsive to rein cues than horses from Australia. This could be an artefact due to the low numbers of respondents from Mexico, Belgium and South Africa (*n* = 19, 18, and 16, respectively).

Beyond such under-representation of certain countries, the authors acknowledge a series of limitations with current data. Due to the large number of independent variables used in our analysis, there was a danger of overfitting the model. To counteract this, we subjected the final model to backwards elimination until all remaining terms were associated with a *p* < 0.2. While it is possible that overfitting has occurred despite the mitigation measure, E-BARQ data collection is ongoing and will allow further validation analyses to be conducted in the future. As with most online surveys, there was the risk of many of the inherent biases [37]. However, the E-BARQ was designed to avoid many such biases. Fundamentally, almost all questions were based on observable behaviours, rather than subjective opinion. However, the items in the Responsiveness index required a degree of interpretation. On a five-point scale ranging from “strongly disagree” to “strongly agree”, respondents were asked if their horse was responsive to rein pressure under different circumstances. Each rider’s definition of a responsive horse may differ depending on their experience or their primary equestrian discipline, making this index susceptible to a degree of confirmation bias [31]. This could also explain why the Responsiveness index and Deceleration index items were loaded onto different rotated components, despite describing similar training cues.

Similarly, questions regarding respondent skill level and experience level were self-reported. There are no universal definitions of rider skill levels [38] and no definition was provided to guide participants in selecting their answer. A separate E-BARQ question asked respondents to select their level of experience with horses. The categories provided ranged from “up to one year’s experience” to “I’ve ridden/handled horses all of my life”. However, it is difficult to interpret these results without further information on the frequency and type of horse-related experience. For example, a year of weekend pleasure riding does not provide the same potential to upskill a rider as a year of intensive instruction would. This means results related to rider skill level and rider experience level are indicative at best. Future research into the effect of beginner riders on ridden horse behaviour should first seek to establish a metric for rider skill level.

As indicated by the findings related to country of residence, E-BARQ data may not represent a true cross section of the equine community. Due to recruitment methods and the length of the questionnaire, E-BARQ is likely to attract respondents with a prior interest in equitation science or equine behaviour. This selection bias may become more pronounced in countries with fewer participants, even though the overall study population (*n* = 1322) may be large enough to be considered representative of the wider community. The E-BARQ continues to gather data and is being translated into Spanish, French, and Italian, so the anticipated increase in sample size will allow the current findings to be more critically examined. Epidemiological investigations, such as this, provide the material for focused hypothesis generation and the elaboration of designed experiments, which can then be used in the pursuit of causation.

## 5. Conclusions

This study found that horses were more difficult to accelerate and less difficult to decelerate when exposed to increasing numbers of riders or handers. The relationship may be due to the inconsistent application of training signals, causing the horse to become habituated, confused, or withdrawn. It may also reflect attempts to match inexperienced riders with a suitably quiet mount. However, riders who considered themselves beginners reported riding horses that were more difficult to both accelerate and decelerate, and less responsive to rein pressure, compared to more experienced riders. This could indicate beginner riders cause the horse to become less responsive to training cues in general. Further longitudinal research is required to reveal the effect of beginner riders on horse behaviour.

## Figures and Tables

**Table 1:** The 10 most common countries of residence for participants of the Equine Behavior Assessment and Research Questionnaire (E-BARQ) survey.

Country	No. of Respondents
Australia	562
United States of America	214
United Kingdom of Great Britain and Northern Ireland	141
Canada	122
New Zealand	112
Mexico	19
Belgium	18
Italy	18
Sweden	17
South Africa	16

**Table 2:** Age range of respondents to the E-BARQ survey.

Age Range	No. of Respondents
Under 18 ^1^	64
18–24 years old	315
25–34 years old	215
35–44 years old	196
45–54 years old	262
55–64 years old	197
65–74 years old	68
75 years or older	3

^1^ Respondents under 18 years of age were instructed to complete the questionnaire under the supervision of a parent or guardian.

**Table 3:** Varimax rotated component loading score for E-BARQ items that loaded in the Acceleration index.

E-BARQ Item	Behaviour	Loading
Q60_13	Responsive to seat cues for upward transitions	−0.44 ^1^
Q57_5	Backs when signalled to move forward	0.46
Q57_6	Not move when signalled with leg or whip cues	0.76
Q57_8	Slow when signalled to go faster	0.69
Q60_4	Responsive to leg pressure to go from walk to trot	−0.67 ^1^
Q60_5	Responsive to leg pressure to go from trot to canter	−0.66 ^1^
Q61_3	Pull behind on lead rope	0.42

^1^ Scores for these items were reversed to ensure uniform scoring within the index.

**Table 4:** Varimax rotated component loading scores for E-BARQ items that loaded strongly in the Deceleration index.

E-BARQ item	Behaviour	Loading
Q57_14	Raise head to avoid rein or lead rope cues	0.68
Q57_15	Toss head when being ridden/driven	0.57
Q57_17	Pull on reins or lead rope when signals are applied	0.71
Q57_18	Brace neck when rein or lead rope signals are applied	0.65
Q57_19	Move faster or raise head when anticipating the transition to canter	0.63
Q57_9	Fail to slow when signalled by a rein or lead rope cue	0.72
Q57_10	Fail to stop when signalled by a rein or lead rope cue	0.71
Q61_1	Pull forward on lead rope ^1^	0.42

^1^ Removal of this item improved Cronbach’s alpha.

**Table 5:** Varimax rotated component loading score for E-BARQ items that loaded in the Responsiveness index.

E-BARQ Item	Behaviour	Loading
Q60_9	Responsive to rein tension to turn	0.78
Q60_12	Responsive to rein tension to slow from canter to trot	0.83
Q60_15	Responsive to whip application (with contact) ^1^	0.58
Q60_11	Responsive to rein tension to slow from walk to halt	0.82

^1^ Removal of this item improved Cronbach’s alpha.

**Table 6:** Breakdown of responses (*n* = 1822) to the E-BARQ question: “Using the past 12 months as a guide, how many different handlers/riders are likely to ride or handle [horse name] per month?”.

Response Selected	No. (%)
I am the only person to ride or handle this horse	659 (36.17%)
2 people	747 (41.00%)
3 people	229 (12.57%)
4 people	97 (5.32%)
5 people	31 (1.70%)
6–10 people	30 (1.65%)
11 or more people	11 (0.60%)
This horse hasn’t been ridden or handled in the last 12 months ^1^	18 (0.99%)

^1^ These horses were excluded from the analysis.

**Table 7:** Univariable analysis of candidate independent variables for inclusion in the final models. Variables with *p* < 0.2 were selected for inclusion. Some demographic variables of interest were forced into the models regardless of *p*-value.

E-BARQ Item	Acceleration		Deceleration		Responsiveness	
	*f-*Value	*p-*Value	*f-*Value	*p-*Value	*f-*Value	*p-*Value
Number of riders	0.3898	0.5325 ^2^	2.8546	0.09131 ^1^	2.5016	0.1139 ^1^
Respondent gender	0.084	0.772 ^2^	6.745	0.009 ^1^	0.151	0.698 ^2^
Country	2.702	0.003 ^1^	2.565	0.004 ^1^	2.228	0.014 ^1^
Respondent age	2.073	0.043 ^1^	3.649	0.001 ^1^	1.508	0.160 ^1^
Horse sex	3.247	0.006 ^1^	1.932	0.103 ^1^	1.502	0.186 ^1^
Horse age	18.683	<0.001 ^1^	10.460	0.001 ^1^	8.658	0.003 ^1^
Horse breed	1.425	0.147 ^1^	5.908	<0.001 ^1^	1.814	0.041 ^1^
Horse discipline	2.503	<0.001 ^1^	2.767	<0.001 ^1^	1.048	0.401
Respondent laterality	0.596	0.551	0.585	0.557	0.584	0.558
Horse colour	1.091	0.365	3.293	<0.001 ^1^	1.648	0.088 ^1^
Frequency of saddle fit evaluations	0.213	0.808	9.883	<0.001 ^1^	0.235	0.791
Experience level of respondent	7.916	<0.001 ^1^	10.003	<0.001 ^1^	3.882	<0.001 ^1^
Skill level of respondent	19.077	<0.001 ^1^	10.018	<0.001 ^1^	7.973	<0.001 ^1^
Summer housing of horse	2.7877	0.007^1^	2.676	0.009 ^1^	1.054	0.3914 ^2^
Winter housing of horse	2.261	0.001	2.017	0.041	0.9515	0.473

^1^ Selected for the final model. ^2^ Forced into the final model.

**Table 8:** Regression coefficients of significant variables (*p* < 0.05) in the Acceleration model. Positive estimates indicate the variable is associated with horses that are harder to accelerate. Negative estimates indicate the variable is associated with horses that are easier to accelerate.

Variable	Estimate	Std. Error	*t-*Value	*p-*Value
Number of riders	0.0148	0.0071	2.0925	0.0366
Horse age	−0.0070	0.0018	−3.8465	0.0001
Country				
Australia (reference)	-	-	-	-
Belgium	−0.2003	0.0700	−2.8611	0.0043
Mexico	−0.3082	0.0688	−4.4813	0.0000
Respondent age				
18–24 years old (reference)	-	-	-	-
45–54 years old	−0.0731	0.0243	−3.0153	0.0026
65–74 years old	−0.1062	0.0387	−2.7487	0.0061
Breed				
Crossbred Horse (reference)	-	-	-	-
Standardbred	0.1002	0.0511	1.9623	0.0499
Discipline				
Pleasure riding (reference)	-	-	-	-
Adult riding club	0.0884	0.0449	1.9670	0.0494
Respondent experience				
All of life (reference)	-	-	-	-
Up to 2 years’ experience	0.1200	0.0564	2.1287	0.0335
More than 8 years’ experience	0.0632	0.0242	2.6156	0.0090
Skill level				
A beginner rider (reference)	-	-	-	-
A novice rider/horse handler	−0.1968	0.0738	−2.6664	0.0078
An intermediate rider/horse handler	−0.2911	0.0745	−3.9058	0.0001
An advanced rider/horse handler	−0.3539	0.0756	−4.6795	0.0000
An elite rider	−0.5349	0.0996	−5.3725	0.0000

**Table 9:** Regression coefficients for significant variables (*p* < 0.05) in the Deceleration model. Variables with a positive estimate are associated with horses that are more difficult to decelerate. Variables with a negative estimate are associated with horses that are less difficult to decelerate.

Variable	Estimate	Std. Error	*t-*Value	*p-*Value
Number of riders	−0.017	0.008	−2.177	0.030
Horse age	−0.005	0.002	−3.031	0.002
Country				
Australia (reference)	-	-	-	-
Belgium	−0.174	0.079	−2.201	0.028
Mexico	−0.202	0.076	−2.661	0.008
Respondent age				
18–24 years old (reference)	-	-	-	-
45–54 years old	−0.080	0.027	−2.996	0.003
55–64 years old	−0.080	0.030	−2.705	0.007
65–74 years old	−0.116	0.043	−2.721	0.007
Breed				
Crossbred horse (reference)	-	-	-	-
Standardbred	−0.122	0.057	−2.160	0.031
Heavy Horse	−0.108	0.052	−2.096	0.036
Iberian	−0.173	0.061	−2.845	0.005
Pony	−0.145	0.057	−2.556	0.011
Warmblood	−0.116	0.034	−3.385	0.001
Quarter Horse	−0.102	0.034	−3.003	0.003
Discipline				
Pleasure Riding (reference)	-	-	-	-
Liberty	−0.306	0.101	−3.019	0.003
Show-jumping	0.098	0.036	2.755	0.006
Saddle fit frequency				
No professional saddle fitting (reference)	-	-	-	-
Does not (yet) wear a saddle	−0.386	0.087	−4.425	0.000
Horse colour				
Bay (reference)	-	-	-	-
Brown	−0.099	0.031	−3.165	0.002
Grey	−0.061	0.029	−2.074	0.038
Experience level				
All my life (reference)	-	-	-	-
Most of my life	0.066	0.022	3.022	0.003
More than 8 years’ experience	0.064	0.027	2.402	0.016
Up to 8 years’ experience	0.175	0.040	4.418	0.000
Up to 5 years’ experience	0.155	0.039	3.949	0.000
Skill level				
Beginner rider (reference)	-	-	-	-
A non-rider/non-horse person	−0.911	0.366	−2.489	0.013
An intermediate rider/horse handler	−0.169	0.083	−2.052	0.040
An advanced rider/horse handler	−0.204	0.084	−2.436	0.015
An elite rider	−0.420	0.110	−3.812	0.000

**Table 10:** Regression coefficients for significant variables (*p* < 0.05) in the Responsiveness model. Variables with a positive estimate are associated with more responsive horses. Variables with a negative estimate are associated with less responsive horses.

Variable	Estimate	Std. Error	*t-*Value	*p-*Value
Horse age	0.009	0.002	3.582	0.000
Country				
Australia (reference)	-	-	-	-
Belgium	0.195	0.095	2.050	0.041
South Africa	−0.292	0.089	−3.280	0.001
Respondent age				
18–14 years old (reference)	-	-	-	-
45–54 years old	0.090	0.033	2.728	0.006
75 years or older	0.653	0.277	2.359	0.018
Horse sex				
Gelding (reference)	-	-	-	-
Mare (female 3 years or over)	0.110	0.051	2.164	0.031
Horse breed				
Crossbreed (reference)	-	-	-	-
Heavy Horse	0.156	0.063	2.484	0.013
Discipline				
Pleasure riding (reference)	-	-	-	-
Endurance	0.182	0.091	1.992	0.047
Experience level				
Ridden all my life (reference)	-	-	-	-
Up to 8 years’ experience	−0.140	0.050	−2.812	0.005
Skill level				
Beginner rider (reference)	-	-	-	-
An intermediate rider/horse handler	0.219	0.108	2.025	0.043
An advanced rider/horse handler	0.300	0.109	2.739	0.006
An elite rider	0.379	0.139	2.728	0.006
Horse sex: age interactions				
Gelding: Horse age (reference)	-	-	-	-
Mare: Horse age	−0.008	0.004	−2.038	0.042

## Data Availability

The data presented in this study are available in Supplementary Materials.

## Supplementary Materials

The following are available online at https://www.mdpi.com/2076-2615/11/1/66/s1, File S1: E-BARQ Questionnaire, Tables S2–S4: Full results of multivariable modelling.
