# Supplementary material for: Equine Responses to Acceleration and Deceleration Cues May Reflect Their Exposure to Multiple Riders
Source: Animals (Basel). 2020 Dec 31;11(1):66. doi: 10.3390/ani11010066 (PMC7823401; doi:10.3390/ani11010066)
Supplement: Supplementary file 1 [file animals-11-00066-s001.zip › Supplementary files/Full tables.docx]

**Table S2.** Regression coefficients of variables in the *acceleration* model. Positive estimates indicate the variable is associated with horses that are harder to accelerate. Negative estimates indicate the variable is associated with horses that are easier to accelerate. Significant results (*p* < 0.05) are shown in bold.

| **Variable** | **Estimate** | **Std. Error** | **t value** | ***p-*value** |
| --- | --- | --- | --- | --- |
| (Intercept) | 2.8909 | 0.1858 | 15.5632 | **0.0000** |
| Number of riders | 0.0148 | 0.0071 | 2.0925 | **0.0366** |
| Horse age | -0.0070 | 0.0018 | -3.8465 | **0.0001** |
| *Respondent gender* |  |  |  |  |
| Female (reference) | - | - | - | - |
| Male | 0.0033 | 0.0438 | 0.0762 | 0.9393 |
| *Country* |  |  |  |  |
| Australia (reference) | - | - | - | - |
| Belgium | -0.2003 | 0.0700 | -2.8611 | **0.0043** |
| Canada | 0.0082 | 0.0285 | 0.2874 | 0.7738 |
| Italy | -0.1142 | 0.0713 | -1.6012 | 0.1096 |
| Mexico | -0.3082 | 0.0688 | -4.4813 | **0.0000** |
| New Zealand | -0.0013 | 0.0274 | -0.0461 | 0.9632 |
| Other | -0.0204 | 0.0344 | -0.5941 | 0.5526 |
| South Africa | -0.0241 | 0.0675 | -0.3571 | 0.7210 |
| Sweden | -0.0867 | 0.0652 | -1.3282 | 0.1843 |
| United Kingdom | -0.0246 | 0.0280 | -0.8758 | 0.3813 |
| United States of America | -0.0089 | 0.0257 | -0.3469 | 0.7288 |
| *Respondent age* |  |  |  |  |
| 18-24 years old (reference) | - | - | - | - |
| 25-34 years old | 0.0081 | 0.0254 | 0.3194 | 0.7495 |
| 35-44 years old | -0.0059 | 0.0271 | -0.2186 | 0.8270 |
| 45-54 years old | -0.0731 | 0.0243 | -3.0153 | **0.0026** |
| 55-64 years old | -0.0177 | 0.0267 | -0.6613 | 0.5085 |
| 65-74 years old | -0.1062 | 0.0387 | -2.7487 | **0.0061** |
| 75 years or older | 0.1628 | 0.1689 | 0.9637 | 0.3354 |
| Under 18 | -0.0448 | 0.0400 | -1.1200 | 0.2629 |
| *Horse sex* |  |  |  |  |
| Gelding (reference) | - | - | - | - |
| Colt (entire male under 3 years) | 0.1676 | 0.2686 | 0.6240 | 0.5327 |
| Filly (female under 3 years) | -2.4062 | 1.2720 | -1.8917 | 0.0587 |
| Mare (female 3 years or over) | -0.0458 | 0.0368 | -1.2456 | 0.2131 |
| Stallion (entire male 3 years or over) | 0.0041 | 0.1572 | 0.0264 | 0.9790 |
| *Breed* |  |  |  |  |
| Crossbred (reference) | - | - | - | - |
| Arabian | -0.0883 | 0.0486 | -1.8167 | 0.0695 |
| Australian Stock Horse | 0.0199 | 0.0515 | 0.3871 | 0.6988 |
| Standardbred | 0.1002 | 0.0511 | 1.9623 | **0.0499** |
| Thoroughbred | 0.0100 | 0.0219 | 0.4576 | 0.6473 |
| Gaited | -0.0331 | 0.0619 | -0.5349 | 0.5928 |
| Heavy Horse | 0.0339 | 0.0459 | 0.7386 | 0.4603 |
| Iberian | 0.0268 | 0.0540 | 0.4972 | 0.6192 |
| Native | -0.0294 | 0.1307 | -0.2250 | 0.8220 |
| Other | -0.0054 | 0.0353 | -0.1534 | 0.8781 |
| Pony | -0.0001 | 0.0510 | -0.0015 | 0.9988 |
| Warmblood | 0.0228 | 0.0310 | 0.7344 | 0.4628 |
| Quarter Horse | -0.0033 | 0.0304 | -0.1087 | 0.9134 |
| *Discipline* |  |  |  |  |
| Pleasure Riding (reference) | - | - | - | - |
| Adult riding club | 0.0884 | 0.0449 | 1.9670 | **0.0494** |
| Breeding conformation | 0.1113 | 0.1477 | 0.7537 | 0.4511 |
| Companion horse | 0.0409 | 0.0629 | 0.6495 | 0.5161 |
| Competitive riding | -0.0179 | 0.0449 | -0.3995 | 0.6896 |
| Dressage | -0.0275 | 0.0250 | -1.1027 | 0.2703 |
| Endurance | 0.0358 | 0.0669 | 0.5357 | 0.5923 |
| Equitation | 0.0006 | 0.0706 | 0.0084 | 0.9933 |
| Eventing | -0.0441 | 0.0309 | -1.4253 | 0.1543 |
| Liberty | -0.1448 | 0.0923 | -1.5676 | 0.1172 |
| Mounted games | -0.0996 | 0.0647 | -1.5392 | 0.1240 |
| Other | -0.0287 | 0.0424 | -0.6767 | 0.4987 |
| Pony Club | -0.0330 | 0.0447 | -0.7374 | 0.4610 |
| Racing | -0.1696 | 0.0946 | -1.7925 | 0.0733 |
| Showjumping | 0.0098 | 0.0321 | 0.3067 | 0.7591 |
| Therapy horse | -0.1836 | 0.0982 | -1.8700 | 0.0617 |
| Trail riding/hacking | 0.0204 | 0.0307 | 0.6634 | 0.5072 |
| Western events | -0.0444 | 0.0463 | -0.9585 | 0.3380 |
| Western games | -0.1473 | 0.0758 | -1.9435 | 0.0522 |
| Working Equitation | -0.1344 | 0.0754 | -1.7825 | 0.0749 |
| Working horse | -0.0843 | 0.0702 | -1.2014 | 0.2298 |
| *Respondent experience* |  |  |  |  |
| Ridden all my life (reference) | - | - | - | - |
| No experience with horses | 0.0472 | 0.2945 | 0.1604 | 0.8726 |
| Up to 1 year's experience | -0.0610 | 0.0820 | -0.7431 | 0.4576 |
| Up to 2 years' experience | 0.1200 | 0.0564 | 2.1287 | **0.0335** |
| Up to 5 years' experience | 0.0111 | 0.0354 | 0.3146 | 0.7531 |
| Up to 8 years' experience | 0.0451 | 0.0360 | 1.2544 | 0.2099 |
| More than 8 years' experience | 0.0632 | 0.0242 | 2.6156 | **0.0090** |
| Most of Life | 0.0333 | 0.0198 | 1.6803 | 0.0931 |
| *Summer housing* |  |  |  |  |
| A communal barn (reference) | - | - | - | - |
| Paddock either at night or day and stable at other times | -0.0266 | 0.1672 | -0.1590 | 0.8737 |
| Paddock or field 24/7 (with access to shelter) | 0.0034 | 0.1663 | 0.0203 | 0.9838 |
| Stabled 24/7 and taken out for riding, lunging or walking in hand | 0.0255 | 0.1814 | 0.1408 | 0.8880 |
| Stabled 24/7 with free 'play' time and ridden/ground work | -0.0901 | 0.1758 | -0.5124 | 0.6085 |
| Stabled 24/7 with time each day to 'play' in an open area | -0.1045 | 0.1807 | -0.5784 | 0.5631 |
| Usually paddocked but occasionally stabled (weather related) | 0.0228 | 0.1674 | 0.1361 | 0.8917 |
| Usually stabled but occasionally paddocked | 0.1080 | 0.1728 | 0.6248 | 0.5322 |
| *Skill level* |  |  |  |  |
| A beginner rider (reference) | - | - | - | - |
| A non-rider/non-horse person | -0.1068 | 0.3333 | -0.3204 | 0.7487 |
| A novice rider/horse handler | -0.1968 | 0.0738 | -2.6664 | **0.0078** |
| An intermediate rider/horse handler | -0.2911 | 0.0745 | -3.9058 | **0.0001** |
| An advanced rider/horse handler | -0.3539 | 0.0756 | -4.6795 | **0.0000** |
| An elite rider | -0.5349 | 0.0996 | -5.3725 | **0.0000** |
| *Interaction terms* |  |  |  |  |
| Gelding: Horse age (reference) | *-* | - | - | - |
| Colt (entire male under 3 years): Horse age | 0.0030 | 0.0436 | 0.0698 | 0.9443 |
| Filly (female under 3 years): Horse age | 1.1472 | 0.6105 | 1.8792 | 0.0604 |
| Mare (female 3 years or over): Horse age | 0.0018 | 0.0029 | 0.6204 | 0.5351 |
| Stallion (entire male 3 years or over): Horse age | -0.0122 | 0.0107 | -1.1398 | 0.2546 |

**Table S3.** Regression coefficients for variables in the *deceleration* model. Significant results (*p* < 0.05) are in bold. Variables with a positive estimate are associated with horses that are more difficult to decelerate. Variables with a negative estimate are associated with horses that are easier to decelerate.

| **Variable** | **Estimate** | **Std. Error** | **t value** | ***p-*value** |
| --- | --- | --- | --- | --- |
| (Intercept) | 2.857 | 0.206 | 13.904 | **0.000** |
| Number of riders | -0.017 | 0.008 | -2.177 | **0.030** |
| Horse age | -0.005 | 0.002 | -3.031 | **0.002** |
| *Respondent gender* |  |  |  |  |
| Female (reference) | - | - | - | - |
| Male | -0.077 | 0.048 | -1.594 | 0.111 |
| *Country* |  |  |  |  |
| Australia | - | - | - | - |
| Belgium | -0.174 | 0.079 | -2.201 | **0.028** |
| Canada | 0.034 | 0.032 | 1.068 | 0.286 |
| Italy | -0.089 | 0.080 | -1.113 | 0.266 |
| Mexico | -0.202 | 0.076 | -2.661 | **0.008** |
| New Zealand | -0.014 | 0.030 | -0.450 | 0.653 |
| Other | -0.028 | 0.038 | -0.736 | 0.462 |
| South Africa | 0.012 | 0.074 | 0.161 | 0.872 |
| Sweden | 0.138 | 0.072 | 1.905 | 0.057 |
| United Kingdom | -0.015 | 0.031 | -0.475 | 0.635 |
| United States of America | -0.014 | 0.029 | -0.493 | 0.622 |
| *Respondent age* |  |  |  |  |
| 18-24 years old (reference) | - | - | - | - |
| 25-34 years old | -0.019 | 0.028 | -0.683 | 0.495 |
| 35-44 years old | -0.038 | 0.030 | -1.268 | 0.205 |
| 45-54 years old | -0.080 | 0.027 | -2.996 | **0.003** |
| 55-64 years old | -0.080 | 0.030 | -2.705 | **0.007** |
| 65-74 years old | -0.116 | 0.043 | -2.721 | **0.007** |
| 75 years or older | 0.247 | 0.186 | 1.331 | 0.184 |
| Under 18 | -0.079 | 0.044 | -1.769 | 0.077 |
| *Horse sex* |  |  |  |  |
| Gelding (reference) | - | - | - | - |
| Colt (entire male under 3 years) | -0.090 | 0.184 | -0.492 | 0.623 |
| Filly (female under 3 years) | -0.098 | 0.115 | -0.853 | 0.394 |
| Mare (female 3 years or over) | 0.007 | 0.017 | 0.417 | 0.676 |
| Stallion (entire male 3 years or over) | -0.153 | 0.096 | -1.597 | 0.111 |
| *Breed* |  |  |  |  |
| Crossbred horse (reference) | - | - | - | - |
| Arabian | -0.023 | 0.054 | -0.435 | 0.664 |
| Australian Stock Horse | -0.067 | 0.057 | -1.192 | 0.233 |
| Standardbred | -0.122 | 0.057 | -2.160 | **0.031** |
| Thoroughbred | -0.008 | 0.025 | -0.311 | 0.756 |
| Gaited | -0.097 | 0.068 | -1.418 | 0.156 |
| Heavy Horse | -0.108 | 0.052 | -2.096 | **0.036** |
| Iberian | -0.173 | 0.061 | -2.845 | **0.005** |
| Native | -0.103 | 0.144 | -0.715 | 0.475 |
| Other | 0.025 | 0.039 | 0.628 | 0.530 |
| Pony | -0.145 | 0.057 | -2.556 | **0.011** |
| Warmblood | -0.116 | 0.034 | -3.385 | **0.001** |
| Quarter Horse | -0.102 | 0.034 | -3.003 | **0.003** |
| *Discipline* |  |  |  |  |
| Pleasure Riding (reference) | - | - | - | - |
| Adult riding club | -0.008 | 0.050 | -0.162 | 0.872 |
| Breeding conformation | 0.200 | 0.161 | 1.243 | 0.214 |
| Companion horse | 0.049 | 0.071 | 0.690 | 0.490 |
| Competitive riding | 0.004 | 0.050 | 0.086 | 0.931 |
| Dressage | 0.016 | 0.028 | 0.565 | 0.572 |
| Endurance | 0.129 | 0.074 | 1.756 | 0.079 |
| Equitation | -0.066 | 0.078 | -0.853 | 0.394 |
| Eventing | 0.021 | 0.034 | 0.627 | 0.531 |
| Liberty | -0.306 | 0.101 | -3.019 | **0.003** |
| Mounted games | 0.030 | 0.071 | 0.425 | 0.671 |
| Other | -0.007 | 0.047 | -0.152 | 0.879 |
| Pony Club | 0.046 | 0.049 | 0.934 | 0.351 |
| Racing | 0.197 | 0.109 | 1.809 | 0.071 |
| Showjumping | 0.098 | 0.036 | 2.755 | **0.006** |
| Therapy horse | -0.183 | 0.116 | -1.583 | 0.114 |
| Trail riding/hacking | 0.011 | 0.034 | 0.327 | 0.744 |
| Western events | -0.074 | 0.051 | -1.446 | 0.148 |
| Western games | -0.020 | 0.085 | -0.230 | 0.818 |
| Working Equitation | 0.085 | 0.083 | 1.030 | 0.303 |
| Working horse | 0.060 | 0.078 | 0.770 | 0.441 |
| *Saddle fit* |  |  |  |  |
| No professional saddle fitting (reference) | - | - | - | - |
| Does not (yet) wear a saddle | -0.386 | 0.087 | -4.425 | **0.000** |
| Saddle fitted professionally | -0.023 | 0.018 | -1.290 | 0.197 |
| *Hose color* |  |  |  |  |
| Bay (reference) | - | - | - | - |
| Black | -0.025 | 0.032 | -0.784 | 0.433 |
| Brown | -0.099 | 0.031 | -3.165 | **0.002** |
| Chestnut | -0.039 | 0.024 | -1.600 | 0.110 |
| Dilution | -0.080 | 0.042 | -1.931 | 0.054 |
| Grey | -0.061 | 0.029 | -2.074 | **0.038** |
| Leopard | -0.049 | 0.072 | -0.687 | 0.492 |
| Palomino | -0.077 | 0.061 | -1.249 | 0.212 |
| Roan | -0.040 | 0.079 | -0.509 | 0.611 |
| White | -0.033 | 0.202 | -0.162 | 0.872 |
| White patterned | -0.053 | 0.034 | -1.571 | 0.116 |
| *Experience level* |  |  |  |  |
| Ridden all my life (reference) | - | - | - | - |
| Most of my life | 0.066 | 0.022 | 3.022 | **0.003** |
| More than 8 years' experience | 0.064 | 0.027 | 2.402 | **0.016** |
| Up to 8 years' experience | 0.175 | 0.040 | 4.418 | **0.000** |
| Up to 5 years' experience | 0.155 | 0.039 | 3.949 | **0.000** |
| Up to 2 years' experience | 0.098 | 0.063 | 1.557 | 0.120 |
| Up to 1 year's experience | 0.152 | 0.090 | 1.681 | 0.093 |
| No experience with horses | 0.182 | 0.323 | 0.563 | 0.574 |
| *Summer housing* |  |  |  |  |
| Communal barn (reference) | - | - | - | - |
| Paddock either at night or day and stable at other times | 0.093 | 0.185 | 0.501 | 0.616 |
| Paddock or field 24/7 (with access to shelter) | 0.062 | 0.184 | 0.335 | 0.738 |
| Stabled 24/7 and taken out for riding, lunging or walking in hand | -0.022 | 0.201 | -0.108 | 0.914 |
| Stabled 24/7 with free 'play' time and ridden/ground work | 0.082 | 0.194 | 0.424 | 0.672 |
| Stabled 24/7 with time each day to 'play' in an open area | 0.081 | 0.199 | 0.409 | 0.683 |
| Usually paddocked but occasionally stabled (weather related) | 0.085 | 0.185 | 0.458 | 0.647 |
| Usually stabled but occasionally paddocked | 0.175 | 0.191 | 0.918 | 0.359 |
| *Skill level* |  |  |  |  |
| Beginner Rider (reference) | - | - | - | - |
| A non-rider/non-horse person | -0.911 | 0.366 | -2.489 | **0.013** |
| A novice rider/horse handler | -0.127 | 0.082 | -1.554 | 0.120 |
| An intermediate rider/horse handler | -0.169 | 0.083 | -2.052 | **0.040** |
| An advanced rider/horse handler | -0.204 | 0.084 | -2.436 | **0.015** |
| An elite rider | -0.420 | 0.110 | -3.812 | **0.000** |

**Table S4.** Regression coefficients for variables in the *Responsiveness* model. Significant results (*p* < 0.05) appear in bold. Variables with a positive estimate are associated with more responsive horses. Variables with a negative estimate are associated with less responsive horses.

| **Variable** | **Estimate** | **Std. Error** | **t value** | ***p-*value** |
| --- | --- | --- | --- | --- |
| (Intercept) | -2.017 | 0.118 | -17.127 | **0.000** |
| Number of riders | 0.010 | 0.010 | 1.037 | 0.300 |
| Horse age | 0.009 | 0.002 | 3.582 | **0.000** |
| *Respondent gender* |  |  |  |  |
| Female (reference) | - | - | - | - |
| Male | -0.066 | 0.060 | -1.107 | 0.268 |
| *Country* |  |  |  |  |
| Australia (reference) | - | - | - | - |
| Belgium | 0.195 | 0.095 | 2.050 | **0.041** |
| Canada | -0.012 | 0.039 | -0.295 | 0.768 |
| Italy | -0.064 | 0.107 | -0.596 | 0.552 |
| Mexico | 0.086 | 0.095 | 0.909 | 0.364 |
| New Zealand | -0.072 | 0.037 | -1.942 | 0.052 |
| Other | 0.013 | 0.046 | 0.279 | 0.780 |
| South Africa | -0.292 | 0.089 | -3.280 | **0.001** |
| Sweden | 0.030 | 0.089 | 0.342 | 0.732 |
| United Kingdom of Great Britain and Northern Ireland | -0.041 | 0.037 | -1.097 | 0.273 |
| United States of America | 0.023 | 0.034 | 0.665 | 0.506 |
| *Respondent age* |  |  |  |  |
| 18-24 years old (reference) | - | - | - | - |
| 25-34 years old | 0.026 | 0.035 | 0.764 | 0.445 |
| 35-44 years old | 0.022 | 0.037 | 0.589 | 0.556 |
| 45-54 years old | 0.090 | 0.033 | 2.728 | **0.006** |
| 55-64 years old | 0.023 | 0.036 | 0.644 | 0.520 |
| 65-74 years old | 0.091 | 0.052 | 1.747 | 0.081 |
| 75 years or older | 0.653 | 0.277 | 2.359 | **0.018** |
| Under 18 - please complete under the supervision of a parent/guardian | 0.054 | 0.055 | 0.988 | 0.323 |
| *Horse sex* |  |  |  |  |
| Gelding (reference) | - | - | - | - |
| Colt (entire male under 3 years) | -0.170 | 0.364 | -0.467 | 0.641 |
| Filly (female under 3 years) | 3.029 | 1.736 | 1.745 | 0.081 |
| Mare (female 3 years or over) | 0.110 | 0.051 | 2.164 | **0.031** |
| Stallion (entire male 3 years or over) | 0.196 | 0.216 | 0.905 | 0.366 |
| *Horse breed* |  |  |  |  |
| Crossbred (reference) | - | - | - | - |
| Arabian | 0.008 | 0.067 | 0.123 | 0.902 |
| Australian Stock Horse | 0.077 | 0.071 | 1.081 | 0.280 |
| Standardbred | 0.023 | 0.070 | 0.327 | 0.744 |
| Thoroughbred | -0.028 | 0.030 | -0.938 | 0.349 |
| Gaited | -0.054 | 0.082 | -0.653 | 0.514 |
| Heavy Horse | 0.156 | 0.063 | 2.484 | **0.013** |
| Iberian | -0.068 | 0.076 | -0.886 | 0.376 |
| Native | -0.172 | 0.179 | -0.964 | 0.335 |
| Other | -0.034 | 0.048 | -0.704 | 0.482 |
| Pony | 0.050 | 0.069 | 0.721 | 0.471 |
| Warmblood | -0.010 | 0.043 | -0.229 | 0.819 |
| Quarter Horse | 0.019 | 0.041 | 0.457 | 0.648 |
| *Discipline* |  |  |  |  |
| Pleasure Riding (reference) | - | - | - | - |
| Adult riding club | 0.025 | 0.062 | 0.413 | 0.680 |
| Breeding conformation | 0.091 | 0.202 | 0.448 | 0.654 |
| Companion horse | -0.090 | 0.085 | -1.054 | 0.292 |
| Competitive riding | 0.002 | 0.062 | 0.034 | 0.973 |
| Dressage | 0.052 | 0.034 | 1.541 | 0.124 |
| Endurance | 0.182 | 0.091 | 1.992 | **0.047** |
| Equitation | -0.005 | 0.094 | -0.050 | 0.960 |
| Eventing | 0.076 | 0.042 | 1.821 | 0.069 |
| Liberty | 0.217 | 0.132 | 1.653 | 0.098 |
| Mounted games | -0.002 | 0.090 | -0.025 | 0.980 |
| Other | 0.019 | 0.058 | 0.326 | 0.744 |
| Pony Club | 0.056 | 0.059 | 0.935 | 0.350 |
| Racing | 0.015 | 0.129 | 0.119 | 0.905 |
| Showjumping | -0.005 | 0.044 | -0.110 | 0.913 |
| Therapy horse | -0.049 | 0.141 | -0.347 | 0.729 |
| Trail riding/hacking | 0.035 | 0.042 | 0.839 | 0.402 |
| Western events | 0.025 | 0.065 | 0.391 | 0.696 |
| Western games | -0.056 | 0.103 | -0.548 | 0.583 |
| Working Equitation | -0.088 | 0.100 | -0.883 | 0.378 |
| Working horse | 0.130 | 0.093 | 1.400 | 0.162 |
| *Experience level* |  |  |  |  |
| Ridden all my life (reference) | - | - | - | - |
| Ridden most of my life | -0.024 | 0.027 | -0.885 | 0.376 |
| More than 8 years' experience | -0.024 | 0.033 | -0.742 | 0.458 |
| Up to 8 years' experience | -0.140 | 0.050 | -2.812 | **0.005** |
| Up to 5 years' experience | -0.014 | 0.048 | -0.286 | 0.775 |
| Up to 2 years' experience | -0.044 | 0.078 | -0.566 | 0.571 |
| Up to 1 year's experience | 0.060 | 0.112 | 0.541 | 0.588 |
| No experience with horses | -0.254 | 0.401 | -0.633 | 0.527 |
| *Skill level* |  |  |  |  |
| Beginner rider (reference) | - | - | - | - |
| A non-rider/non-horse person | 0.049 | 0.457 | 0.107 | 0.914 |
| A novice rider/horse handler | 0.184 | 0.107 | 1.712 | 0.087 |
| An intermediate rider/horse handler | 0.219 | 0.108 | 2.025 | **0.043** |
| An advanced rider/horse handler | 0.300 | 0.109 | 2.739 | **0.006** |
| An elite rider | 0.379 | 0.139 | 2.728 | **0.006** |
| *Horse sex: age interactions* |  |  |  |  |
| Gelding: Horse age (reference) | - | - | - | - |
| Colt (entire male under 3 years): Horse age | -0.008 | 0.059 | -0.135 | 0.892 |
| Filly (female under 3 years): Horse age | -1.452 | 0.833 | -1.743 | 0.082 |
| Mare (female 3 years or over): Horse age | -0.008 | 0.004 | -2.038 | **0.042** |
| Stallion (entire male 3 years or over): Horse age | -0.003 | 0.015 | -0.221 | 0.825 |
