# Supplementary material for: Equine Responses to Acceleration and Deceleration Cues May Reflect Their Exposure to Multiple Riders
Source: Animals (Basel). 2020 Dec 31;11(1):66. doi: 10.3390/ani11010066 (PMC7823401; doi:10.3390/ani11010066)
Supplement: Supplementary file 1 [file animals-11-00066-s001.zip › Supplementary files/Data analysis.pdf]

Data Handling and analysis

Project: How does the number of riders affect the horse's responsiveness to acceleration and deceleration signals

Main Dependent variables of interest:

- E1 (5 EBARQ items)
- E3 (6 EBARQ items)
- E5 (3 EBARQ items)
- E8 (5 EBARQ items)
- E11 (2 EBARQ items)
- Seat cues for downwards transitions (unloaded)

Main Predictor variable of interest:

- Q40: Using the past 6 months as a guide, how many different handlers/riders are likely to ride or handle [Field-horsename] per month?

Dependent Variables

Determination of number and composition of Dependent Indicies

Information from 5 EBARQ components (Totalling 21 EBARQ items), and 1 further unloaded item was considered.

To combine these into a smaller number of relatively uncorrelated indices, a Parallel Analysis, comparing the scree of components of the standardised observed data with that of a random data matrix of the same size, was used to assess the underlying number of factors in the 21 items using the psych package of R statistical software .

This Parallel Analysis suggested 4 underlying components with loadings as follows.

EBARQ ITEM	Preliminary analysis components	RC1	RC2	RC3	RC4	EBARQ question
Q60_13seat_speedEquitation	E unloaded	-0.05	0.31	-0.44	-0.05	When ridden or driven, [Field-horsename] is responsive to: - Seat cues for upward transitions
Q57_14raise_headEquitation	E1	0.68	-0.03	0.15	-0.06	Does [Field-horsename]? - Raise head to avoid rein or lead rope cues
Q57_15toss_headEquitation	E1	0.57	0.01	0.21	0.03	Does [Field-horsename]? - Toss head when being ridden/driven
Q57_17pull_on_reinsEquitation	E1	0.71	-0.04	0.17	-0.09	Does [Field-horsename]? - Pull on reins or lead rope when signals are applied
Q57_18brace_neckEquitation	E1	0.65	-0.04	0.17	-0.1	Does [Field-horsename]? - Brace neck when rein or lead rope signals are applied
Q57_19excited_canterEquitation	E1	0.63	-0.06	0.12	0.01	Does [Field-horsename]? - Move faster or raise head when anticipating the transition to canter
Q57_5back_not_forwardEquitation	E11	0.22	0.06	0.46	0.02	Does [Field-horsename]? - Back when signaled to move forward
Q57_6no_forwardEquitation	E11	0.14	-0.01	0.76	-0.02	Does [Field-horsename]? - Not move when signaled with leg or whip cues

Q57_8slows_when_asked_fastEquitation	E3	0.08	-0.01	0.69	0.02	Does [Field-horsename]? - Slow when signaled to go faster
Q60_4leg_pressure_walk_trotEquitation	E3	0.06	0.44	-0.67	0.01	When ridden or driven, [Field-horsename] is responsive to: - Leg pressure to go from walk to trot
Q60_5leg_trot_canterEquitation	E3	0.01	0.41	-0.66	0	When ridden or driven, [Field-horsename] is responsive to: - Leg pressure to go from trot to canter
Q60_9rein_turnEquitation	E3	-0.07	0.78	-0.11	0	When ridden or driven, [Field-horsename] is responsive to: - Rein tension to turn
Q60_12rein_slowEquitation	E3	-0.21	0.83	-0.01	-0.01	When ridden or driven, [Field-horsename] is responsive to: - Rein tension to slow from canter to trot
Q60_15whipEquitation	E3	0.03	0.58	-0.26	-0.02	When ridden or driven, [Field-horsename] is responsive to: - Whip application (with contact)
Q57_9no_slowEquitation	E5	0.72	-0.21	-0.09	0.07	Does [Field-horsename]? - Fail to slow when signaled by a rein or lead rope cue
Q57_10no_stopEquitation	E5	0.71	-0.22	-0.04	0.09	Does [Field-horsename]? - Fail to stop when signaled by a rein or lead rope cue
Q60_11rein_tension_halt Equitation	E5	-0.22	0.82	-0.01	0.04	When ridden or driven, [Field-horsename] is responsive to: - Rein tension to slow from walk to halt
Q61_1	E8	0.42	-0.01	0.03	0.17	When on a lead rope in a familiar or typical situation, does [Field-horsename] pull: - Forward when walking
Q61_3	E8	0.13	-0.02	0.42	0	When on a lead rope in a familiar or typical situation, does [Field-horsename] pull: - Behind (lagging)
Q74_16	E8	0.13	0.01	0.07	0.75	Does [Field-horsename]? - Kick when travelling in the trailer
Q74_17	E8	0.16	0.02	0.04	0.77	Does [Field-horsename]? - Vocalize when travelling in the trailer
Q61_2	E8	0.07	0.01	0.02	-0.16	When on a lead rope in a familiar or typical situation, does [Field-horsename] pull: - Forward when trotting

Only three of the varimax rotated components (RC1, RC2 and RC3 were directly deemed relevant to the research question, so RC4 was not studied further. It is worth noting that some EBARQ items loaded considerably onto more than one component, although none loaded strongly >0.50, onto more than 1.

Cronbach's alpha was used to optimise which items were included in each index. Alpha was calculated for each index including all terms loading more than 0.40 on that rotated component (provided it did not load more strongly onto another component), and terms were removed if the removal improved the value of alpha.

Construction of Dependent Indices

Acceleration

Candidate traits for this index are shown below.

EBARQ ITEM	Prelim	RC3	
Q60_13seat_speedEquitation	E unloaded	-0.44	When ridden or driven, [Field-horsename] is responsive to: - Seat cues for upward transitions
Q57_5back_not_forwardEquitation	E11	0.46	Does [Field-horsename]? - Back when signaled to move forward
Q57_6no_forwardEquitation	E11	0.76	Does [Field-horsename]? - Not move when signaled with leg or whip cues
Q57_8slows_when_asked_fastEquitation	E3	0.69	Does [Field-horsename]? - Slow when signaled to go faster

Q60_4leg_pressure_walk_trotEquitation	E3	-0.67	When ridden or driven, [Field-horsename] is responsive to: - Leg pressure to go from walk to trot
Q60_5leg_trot_canterEquitation	E3	-0.66	When ridden or driven, [Field-horsename] is responsive to: - Leg pressure to go from trot to canter
Q61_3	E8	0.42	When on a lead rope in a familiar or typical situation, does [Field-horsename] pull: - Behind (lagging)

The alpha for these seven items 0.72 (95% CI 0.70-0.74). The removal of no single item resulted in a higher alpha.

Deceleration

Candidate traits for this index are shown below.

EBARQ ITEM	Prelim	RC1	
Q57_14raise_headEquitation	E1	0.68	Does [Field-horsename]? - Raise head to avoid rein or lead rope cues
Q57_15toss_headEquitation	E1	0.57	Does [Field-horsename]? - Toss head when being ridden/driven
Q57_17pull_on_reinsEquitation	E1	0.71	Does [Field-horsename]? - Pull on reins or lead rope when signals are applied
Q57_18brace_neckEquitation	E1	0.65	Does [Field-horsename]? - Brace neck when rein or lead rope signals are applied
Q57_19excited_canterEquitation	E1	0.63	Does [Field-horsename]? - Move faster or raise head when anticipating the transition to canter
Q57_9no_slowEquitation	E5	0.72	Does [Field-horsename]? - Fail to slow when signaled by a rein or lead rope cue
Q57_10no_stopEquitation	E5	0.71	Does [Field-horsename]? - Fail to stop when signaled by a rein or lead rope cue
Q61_1	E8	0.42	When on a lead rope in a familiar or typical situation, does [Field-horsename] pull: - Forward when walking

The alpha for these eight items was 0.80 (95% CI 0.79-0.72), however the removal of item Q61_1 raised the alpha to 0.81 (95% CI 0.80-0.82). Therefore only the other seven items were used to construct this index.

Responsiveness

Candidate traits for this index were

EBARQ ITEM	Prelim	RC2	
Q60_9rein_turnEquitation	E3	0.78	When ridden or driven, [Field-horsename] is responsive to: - Rein tension to turn
Q60_12rein_slowEquitation	E3	0.83	When ridden or driven, [Field-horsename] is responsive to: - Rein tension to slow from canter to trot
Q60_15whipEquitation	E3	0.58	When ridden or driven, [Field-horsename] is responsive to: - Whip application (with contact)
Q60_11rein_tension_haltEquitation	E5	0.82	When ridden or driven, [Field-horsename] is responsive to: - Rein tension to slow from walk to halt

The alpha for these four items was 0.78 (95% CI 0.76-0.80). however the removal of item Q60_15whipEquitation raised the alpha to 0.83 (95% CI 0.82-0.85). Therefore only the other three items were used to construct this index.

Construction of Dependent Indices

Acceleration, Deceleration and Responsiveness indices were constructed by assigning a numerical value to scores on the Likert scale of the relevant EBARQ items, and summing these values together.

In the case of missing values, the sum was divided by the number of EBARQ items in the index for which information for that horse was available and multiplied by the number of items used to calculate the index, weighting the missing value according to the horse's score for similar items rather than imputing an overall mean. If no EBARQ items for an index were completed then a value for that horse was not calculated.

Numerical values were assigned as follows, Never=1, Rarely=2, Sometimes=3, Usually=4, Always =5 or Strongly disagree=1 , Disagree=2,Neutral=3, Agree=4,Strongly agree=5, apart from item Q60_13seat_speedEquitation, Q57_8slows_when_asked_fastEquitation and Q60_4leg_pressure_walk_trotEquitation which was assigned Strongly disagree=5 , Disagree=4,Neutral=3, Agree=2,Strongly agree=1 so that these correlated positively with the remaining items in the Index.

<u>Boxplots of Indices</u>

Boxplots showing the range and distribution of these Indices are shown below.

Acceleration

Composed of seven traits with five levels, this index has a hypothetical range of 7 to 35.

The distribution may be seen below

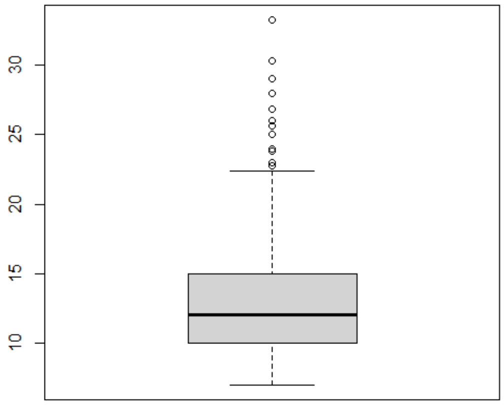

Log transformation of this index corrects negative skew in the index scores, and therefore might be expected to do so in the residuals following analysis.

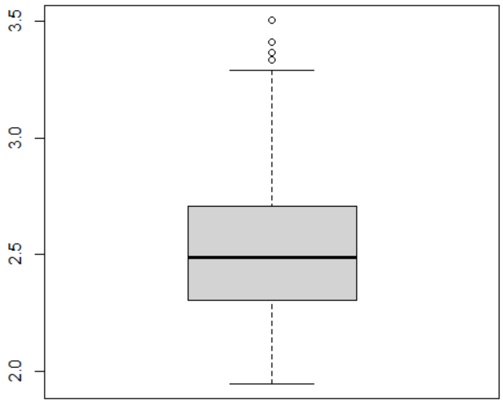

Deceleration

Composed of seven traits with five levels, this index has a hypothetical range of 7 to 35.

The distribution may be seen below

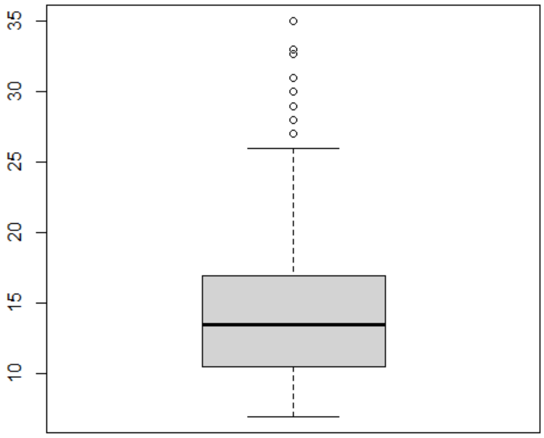

Log transformation of this index corrects negative skew in the index scores, and therefore might be expected to do so in the residuals following analysis.

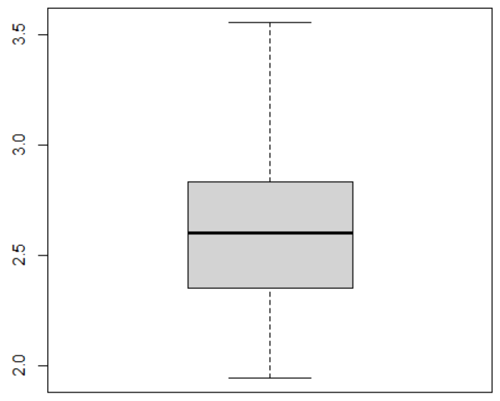

Responsiveness

Composed of three traits with five levels, this index has a hypothetical range of 3 to 15.

The distribution may be seen below

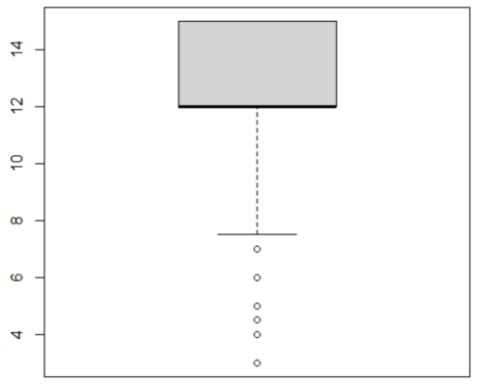

Unlike the prior indices, this index has a negative skew.

Correcting the skew will involve reverting the scores (18-score; so a score of 3 becomes one of 15, and vice versa,) taking the square root of the score, and then reversing the direction again (say by multiplying by -1) so that it remains an index of sensitivity rather than one of insensitivity.

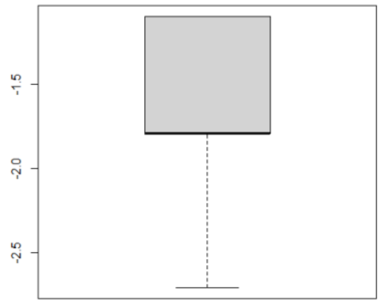

Note that this score is the least normal appearing so inspecting the residuals will be particularly important for this index.

Independent Variables

The main predictor variable of interest is Q40: Using the past 6 months as a guide, how many different handlers/riders are likely to ride or handle [Field-horsename] per month?

This variable will be forced into all models

The data was supplied in an uneven ordinal fashion as shown.

I am the only person to ride or handle this horse	549
2 people	670
3 people	217
4 people	85
5 people	30
6 - 10 people	28
11 or more people	12

There are many possible ways to code this variable. We will treat it as an ordered variable with

[1]= "I am the only person to ride or handle this horse"

[2] ="2 people"

[3] ="3 people"

[4] ="4 people"

[5] ="5 people"

[6] ="6 + people"

Other predictor variables

Basic demographic variables (Rider Gender, Country, Rider Age, Horse age, Horse Sex, Breed and Discipline were forced into the model.

A variable describing housing was included. Because both winter and summer housing was included and to avoid collinearity, Summer housing was selected at random using the sampling function from the base package of r.

Other predictor variables requested were assessed for potential inclusion in the final model by univariable analysis of the indices, transformed as above. Those with P<0.2 were selected.

	Hard to Start		Hard to Stop		Sensitive to Cues	
	F value	P value	F value	P value	F value	P value
N_riders	0.3898	0.5325	2.8546	0.09131	2.5016	0.1139
Gender	0.084	0.772	6.745	0.009	0.151	0.698
Analysis_Country	2.702	0.003	2.565	0.004	2.228	0.014
Age	2.073	0.043	3.649	0.001	1.508	0.160
Sex_horse	3.247	0.006	1.932	0.103	1.502	0.186
Age_horse	18.683	<0.001	10.460	0.001	8.658	0.003
Analysis_breed	1.425	0.147	5.908	<0.001	1.814	0.041
Analysis_Discipline	2.503	<0.001	2.767	<0.001	1.048	0.401
Laterality	0.596	0.551	0.585	0.557	0.584	0.558
Analysis_colour	1.091	0.365	3.293	<0.001	1.648	0.088
Saddle_Fit	0.213	0.808	9.883	<0.001	0.235	0.791
Experience	7.916	<0.001	10.003	<0.001	3.882	<0.001
Self_Eval	19.077	<0.001	10.018	<0.001	7.973	<0.001
Q54	2.7877	0.007	2.676	0.009	1.054	0.3914
Q56	2.261	0.001	2.017	0.041	0.9515	0.473

Multivariate modelling

The Indices were subjected to multivariable analysis. The forced variables and other variables selected from univariable analysis were included in the full model. The least significant optional term was then removed until, all optional terms were p>0.2.

Rider Gender by Age interaction, Horse sex by horse age interactions and Horse age by Breed interactions were added individually to the Final model and retained if P<0.2.

Acceleration index

```
modFinal <- lm(log(mydata$StartHARD) ~ N_riders2+
                    Gender+
                    Analysis_Country+
                    Age+
                    Sex_horse+
                    Age_horse+
                    Analysis_breed+
                    Analysis_Discipline+
```

Experience+
Q54+
Sex_horse:Age_horse+ (sex of horse and age of horse interaction)
Self_Eval, data = mydata)

Model Diagnostics

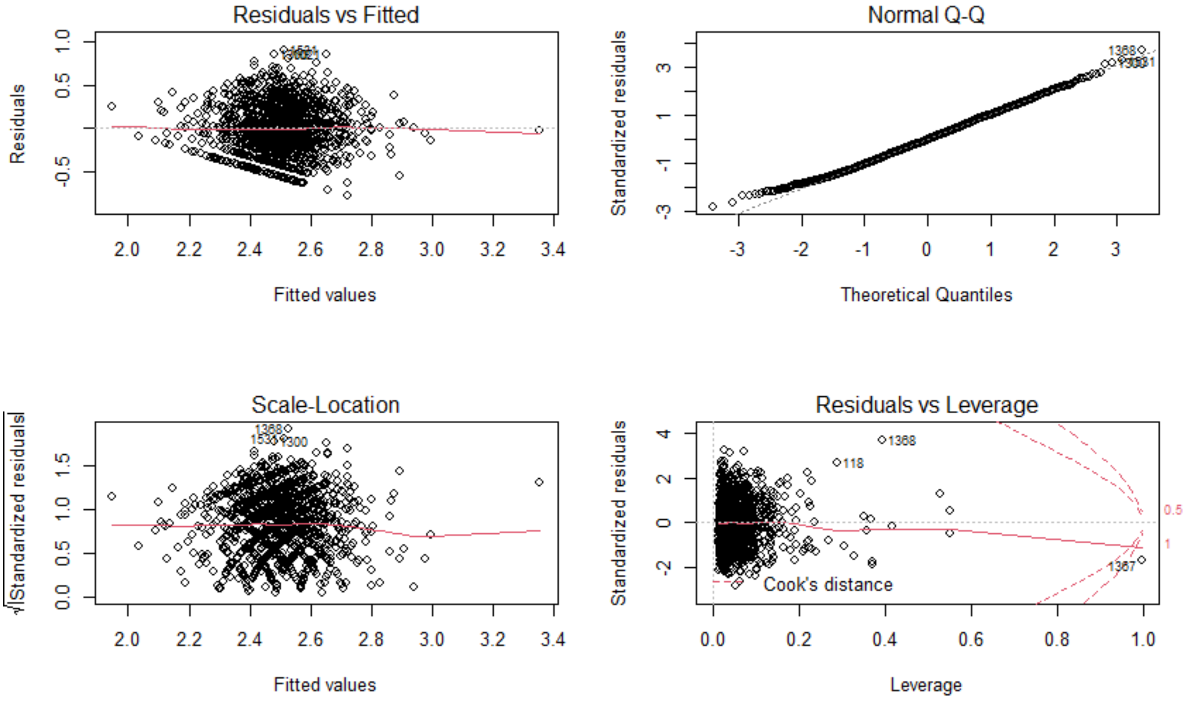

The cooks distance is way out there on that one observation, so lets run it again without it.

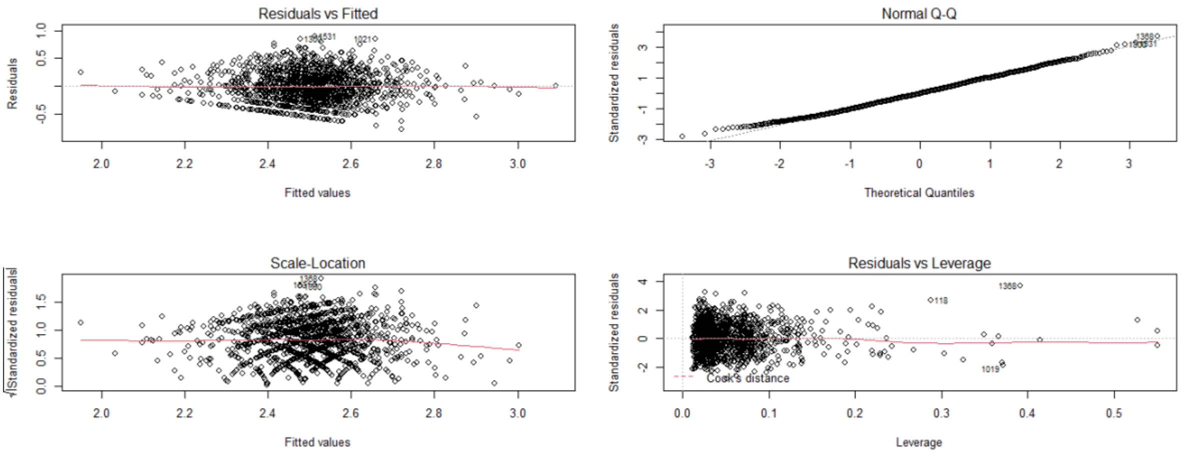

That's better!

We’ll now check the variance inflation factors (we have to take the interaction term out)

	GVIF	Df	GVIF^(1/(2*Df))	Square of GVIF^(1/(2*Df))
N_riders2	1.16881	1	1.081115	1.168809643
Gender	1.285068	1	1.133608	1.285067098
Analysis_Country	4.819019	10	1.081802	1.170295567
Age	2.609799	7	1.070922	1.14687393
Sex_horse	1.482736	4	1.050468	1.103483019
Age_horse	1.136736	1	1.066178	1.136735528
Analysis_breed	4.980014	12	1.069181	1.143148011
Analysis_Discipline	10.21449	20	1.059816	1.123209954
Experience	3.275642	7	1.088446	1.184714695
Q54	2.346852	7	1.062829	1.129605483
Self_Eval	3.490518	5	1.133154	1.284037988

This looks fine too.

So RESULTS!!!

Model fit results

Residual standard error: 0.2792 on 1377 degrees of freedom
(142 observations deleted due to missingness)
Multiple R-squared: 0.1549, Adjusted R-squared: 0.1064
F-statistic: 3.195 on 79 and 1377 DF, p-value: < 2.2e-16

Analysis of Variance type 3 table

	Sum Sq	Df	F value	Pr(>F)
(Intercept)	18.875	1	242.2146	< 2.2e-16
N_riders2	0.341	1	4.3787	0.0366
Gender	0	1	0.0058	0.9393
Analysis_Country	2.486	10	3.1907	0.0005
Age	1.671	7	3.0636	0.0033
Sex_horse	0.436	4	1.3993	0.2319
Age_horse	1.153	1	14.7956	0.0001
Analysis_breed	0.76	12	0.8132	0.6371
Analysis_Discipline	2.399	20	1.5394	0.0600
Experience	0.973	7	1.7832	0.0867
Q54	0.957	7	1.7544	0.0927
Self_Eval	3.718	5	9.5414	0.0000
Sex_horse:Age_horse	0.419	4	1.3444	0.2513
Residuals	107.304	1377		

Regression Coefficients

	Estimate	Std. Error	t value	Pr(>|t|)
(Intercept)	2.8909	0.1858	15.5632	0.0000
N_riders2	0.0148	0.0071	2.0925	0.0366
ref = Female				
GenderMale	0.0033	0.0438	0.0762	0.9393
ref=Australia				
Analysis_CountryBelgium	-0.2003	0.0700	-2.8611	0.0043
Analysis_CountryCanada	0.0082	0.0285	0.2874	0.7738
Analysis_CountryItaly	-0.1142	0.0713	-1.6012	0.1096
Analysis_CountryMexico	-0.3082	0.0688	-4.4813	0.0000
Analysis_CountryNew Zealand	-0.0013	0.0274	-0.0461	0.9632
Analysis_CountryOther	-0.0204	0.0344	-0.5941	0.5526
Analysis_CountrySouth Africa	-0.0241	0.0675	-0.3571	0.7210
Analysis_CountrySweden	-0.0867	0.0652	-1.3282	0.1843
Analysis_CountryUnited Kingdom	-0.0246	0.0280	-0.8758	0.3813
Analysis_CountryUnited States of America	-0.0089	0.0257	-0.3469	0.7288
ref= 18-24				
Age25-34 years old	0.0081	0.0254	0.3194	0.7495
Age35-44 years old	-0.0059	0.0271	-0.2186	0.8270
Age45-54 years old	-0.0731	0.0243	-3.0153	0.0026
Age55-64 years old	-0.0177	0.0267	-0.6613	0.5085
Age65-74 years old	-0.1062	0.0387	-2.7487	0.0061
Age75 years or older	0.1628	0.1689	0.9637	0.3354
AgeUnder 18 - please complete under the supervision of a parent/guardian	-0.0448	0.0400	-1.1200	0.2629
ref=Gelding				
Sex_horseColt (entire male under 3 years)	0.1676	0.2686	0.6240	0.5327
Sex_horseFilly (female under 3 years)	-2.4062	1.2720	-1.8917	0.0587
Sex_horseMare (female 3 years or over)	-0.0458	0.0368	-1.2456	0.2131
Sex_horseStallion (entire male 3 years or over)	0.0041	0.1572	0.0264	0.9790
Age_horse	-0.0070	0.0018	-3.8465	0.0001
ref= Crossbred horse				
Analysis_breed Arabian	-0.0883	0.0486	-1.8167	0.0695
Analysis_breed Australian Stock Horse	0.0199	0.0515	0.3871	0.6988
Analysis_breed Standardbred	0.1002	0.0511	1.9623	0.0499
Analysis_breed Thoroughbred	0.0100	0.0219	0.4576	0.6473
Analysis_breedGaited	-0.0331	0.0619	-0.5349	0.5928
Analysis_breedHeavyHorse	0.0339	0.0459	0.7386	0.4603
Analysis_breedIberian	0.0268	0.0540	0.4972	0.6192
Analysis_breedNative	-0.0294	0.1307	-0.2250	0.8220
Analysis_breedOther	-0.0054	0.0353	-0.1534	0.8781
Analysis_breedPonyGroup	-0.0001	0.0510	-0.0015	0.9988
Analysis_breedWarmblood	0.0228	0.0310	0.7344	0.4628

Analysis_breedWestern_QuarterHorse	-0.0033	0.0304	-0.1087	0.9134
ref= Pleasure Riding				
Analysis_DisciplineAdult riding club	0.0884	0.0449	1.9670	0.0494
Analysis_DisciplineBreeding_conformation	0.1113	0.1477	0.7537	0.4511
Analysis_DisciplineCompanion horse	0.0409	0.0629	0.6495	0.5161
Analysis_DisciplineCompetitive_riding	-0.0179	0.0449	-0.3995	0.6896
Analysis_DisciplineDressage	-0.0275	0.0250	-1.1027	0.2703
Analysis_DisciplineEndurance	0.0358	0.0669	0.5357	0.5923
Analysis_DisciplineEquitation	0.0006	0.0706	0.0084	0.9933
Analysis_DisciplineEventing	-0.0441	0.0309	-1.4253	0.1543
Analysis_DisciplineLiberty	-0.1448	0.0923	-1.5676	0.1172
Analysis_DisciplineMounted_games	-0.0996	0.0647	-1.5392	0.1240
Analysis_DisciplineOther	-0.0287	0.0424	-0.6767	0.4987
Analysis_DisciplinePony Club	-0.0330	0.0447	-0.7374	0.4610
Analysis_DisciplineRacing	-0.1696	0.0946	-1.7925	0.0733
Analysis_DisciplineShow-jumping	0.0098	0.0321	0.3067	0.7591
Analysis_DisciplineTherapy_horse	-0.1836	0.0982	-1.8700	0.0617
Analysis_DisciplineTrail riding/hacking	0.0204	0.0307	0.6634	0.5072
Analysis_DisciplineWestern_events	-0.0444	0.0463	-0.9585	0.3380
Analysis_DisciplineWestern_games	-0.1473	0.0758	-1.9435	0.0522
Analysis_DisciplineWorking Equitation	-0.1344	0.0754	-1.7825	0.0749
Analysis_DisciplineWorking_horse	-0.0843	0.0702	-1.2014	0.2298
ref= ALL of life				
ExperienceNo experience with horses	0.0472	0.2945	0.1604	0.8726
ExperienceUp to 1 year's experience	-0.0610	0.0820	-0.7431	0.4576
ExperienceRider/handler with up to 2 years' experience	0.1200	0.0564	2.1287	0.0335
ExperienceRider/handler with up to 5 years' experience	0.0111	0.0354	0.3146	0.7531
ExperienceRider/handler with up to 8 years' experience	0.0451	0.0360	1.2544	0.2099
ExperienceRider/handler with more than 8 years' experience	0.0632	0.0242	2.6156	0.0090
Experience Most of Life	0.0333	0.0198	1.6803	0.0931
ref= "A communal barn"				
Q54Paddock either at night or day and stable at other times	-0.0266	0.1672	-0.1590	0.8737
Q54Paddock or field 24/7 (with access to shelter)	0.0034	0.1663	0.0203	0.9838
Q54Stabled 24/7 and taken out for riding, lunging or walking in hand	0.0255	0.1814	0.1408	0.8880
Q54Stabled 24/7 with free 'play' time and ridden/ground work	-0.0901	0.1758	-0.5124	0.6085
Q54Stabled 24/7 with time each day to 'play' in an open area	-0.1045	0.1807	-0.5784	0.5631
Q54Usually paddocked but occasionally stabled (weather related)	0.0228	0.1674	0.1361	0.8917
Q54Usually stabled but occasionally paddocked	0.1080	0.1728	0.6248	0.5322
ref= a beginner rider				
Self_EvalA non-rider/non-horse person	-0.1068	0.3333	-0.3204	0.7487
Self_EvalA novice rider/horse handler	-0.1968	0.0738	-2.6664	0.0078
Self_EvalAn intermediate rider/horse handler	-0.2911	0.0745	-3.9058	0.0001
Self_EvalAn advanced rider/horse handler	-0.3539	0.0756	-4.6795	0.0000
Self_EvalAn elite rider	-0.5349	0.0996	-5.3725	0.0000

Interaction terms				
Sex_horseColt (entire male under 3 years):Age_horse	0.0030	0.0436	0.0698	0.9443
Sex_horseFilly (female under 3 years):Age_horse	1.1472	0.6105	1.8792	0.0604
Sex_horseMare (female 3 years or over):Age_horse	0.0018	0.0029	0.6204	0.5351
Sex_horseStallion (entire male 3 years or over):Age_horse	-0.0122	0.0107	-1.1398	0.2546

Partial residual plot for Acceleration

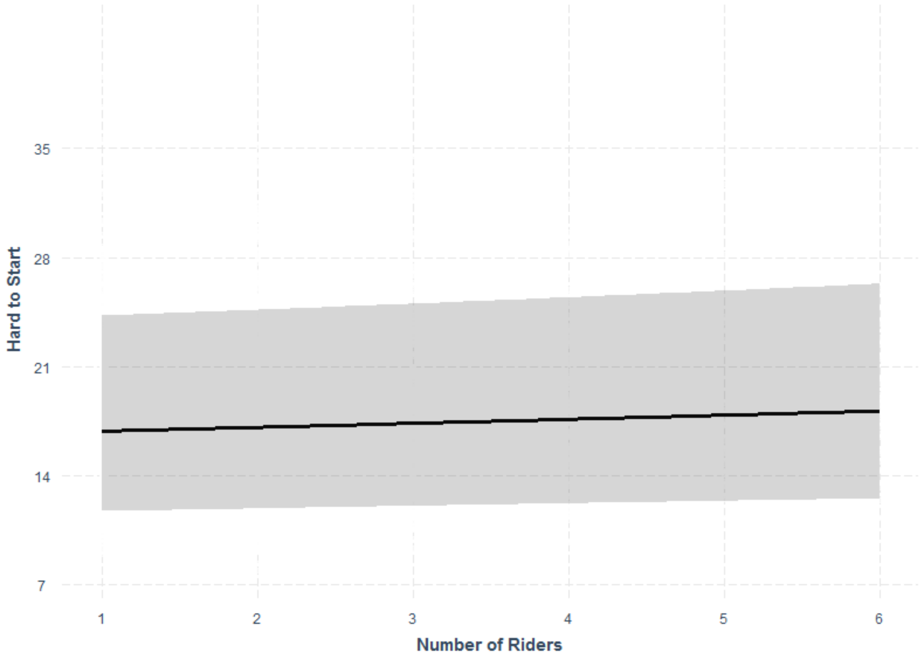

Deceleration Index

```
ModFinal <- lm(log(mydata$StopHARD) ~ N_riders2+
                        Gender+
                        Analysis_Country+
                        Age+
                        Sex_horse+
                        Age_horse+
                        Analysis_breed+
                        Analysis_Discipline+
                        Saddle_Fit+
                        Analysis_colour+
                        Experience+
                        Q54+
                        Self_Eval, data = mydata)
```

Model Diagnostics

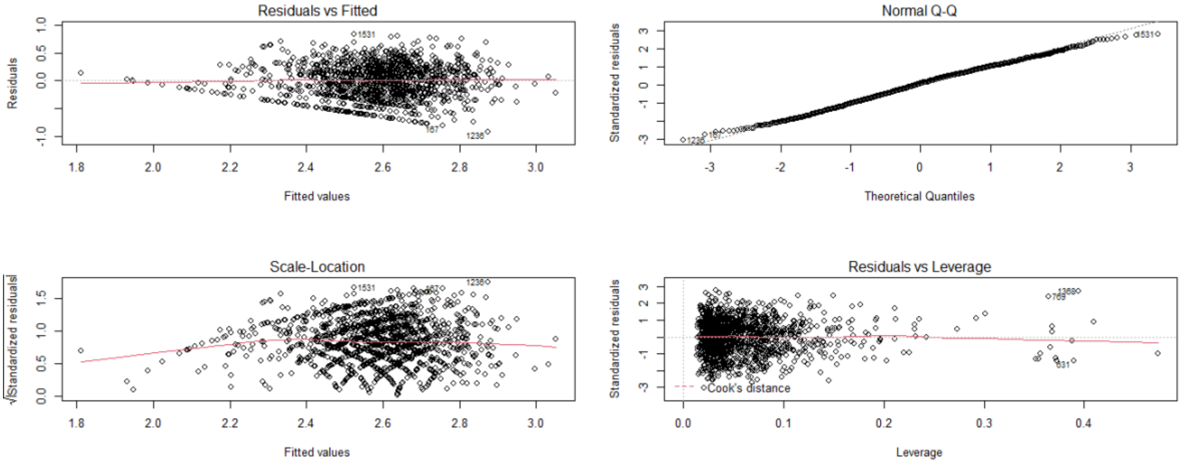

These look acceptable I think.

We'll now check the variance inflation factors (no interaction term to take out this time!)

	GVIF	Df	GVIF^(1/(2*Df))	Square of GVIF^(1/(2*Df))
N_riders2	1.174456	1	1.083723	1.174455541
Gender	1.304901	1	1.142323	1.304901836
Analysis_Country	5.619464	10	1.090146	1.188418301
Age	2.835358	7	1.077281	1.160534353
Sex_horse	1.66226	4	1.065583	1.13546713
Age_horse	1.163025	1	1.078436	1.163024206
Analysis_breed	7.187052	12	1.085649	1.178633751
Analysis_Discipline	15.90326	20	1.071611	1.148350135
Saddle_Fit	1.329607	2	1.073818	1.153085097
Analysis_colour	3.48601	10	1.064428	1.133006967
Experience	3.498779	7	1.093582	1.195921591
Q54	2.592438	7	1.070411	1.145779709
Self_Eval	3.523089	5	1.134207	1.286425519

No problem detected here.

So RESULTS!!!

<u>Model fit results</u>

Residual standard error: 0.3059 on 1361 degrees of freedom
(151 observations deleted due to missingness)
Multiple R-squared: 0.1887, Adjusted R-squared: 0.1368
F-statistic: 3.638 on 87 and 1361 DF, p-value: < 2.2e-16

Analysis of Variance type 2 table (type 2 in this case because no interaction term)

	Sum Sq	Df	F value	Pr(>F)
(Intercept)	18.09	1	193.3317	< 2.2e-16
N_riders2	0.443	1	4.7381	0.0296735
Gender	0.238	1	2.5399	0.1112314
Analysis_Country	1.797	10	1.92	0.038738
Age	1.822	7	2.7813	0.0071127
Sex_horse	0.365	4	0.9753	0.419928
Age_horse	0.86	1	9.1884	0.0024816
Analysis_breed	3.665	12	3.2638	0.0001155
Analysis_Discipline	3.321	20	1.7745	0.0187275
Saddle_Fit	1.92	2	10.2611	3.78E-05
Analysis_colour	1.309	10	1.3994	0.1746603
Experience	2.601	7	3.9706	0.0002636
Q54	0.636	7	0.9717	0.4502781
Self_Eval	2.192	5	4.6846	0.0003024
Residuals	127.349	1361		

Regression Coefficients

	Estimate	Std. Error	t value	Pr(>|t|)
(Intercept)	2.857	0.206	13.904	0.000
N_riders2	-0.017	0.008	-2.177	0.030
ref =Female				
GenderMale	-0.077	0.048	-1.594	0.111
Ref=Australia				
Analysis_CountryBelgium	-0.174	0.079	-2.201	0.028
Analysis_CountryCanada	0.034	0.032	1.068	0.286
Analysis_CountryItaly	-0.089	0.080	-1.113	0.266
Analysis_CountryMexico	-0.202	0.076	-2.661	0.008
Analysis_CountryNew Zealand	-0.014	0.030	-0.450	0.653
Analysis_CountryOther	-0.028	0.038	-0.736	0.462
Analysis_CountrySouth Africa	0.012	0.074	0.161	0.872
Analysis_CountrySweden	0.138	0.072	1.905	0.057
Analysis_CountryUnited Kingdom of Great Britain and Northern Ireland	-0.015	0.031	-0.475	0.635
Analysis_CountryUnited States of America	-0.014	0.029	-0.493	0.622
Ref= 18-24 years old				
Age25-34 years old	-0.019	0.028	-0.683	0.495
Age35-44 years old	-0.038	0.030	-1.268	0.205
Age45-54 years old	-0.080	0.027	-2.996	0.003
Age55-64 years old	-0.080	0.030	-2.705	0.007
Age65-74 years old	-0.116	0.043	-2.721	0.007
Age75 years or older	0.247	0.186	1.331	0.184

AgeUnder 18 - please complete under the supervision of a parent/guardian	-0.079	0.044	-1.769	0.077
Ref= Gelding				
Sex_horseColt (entire male under 3 years)	-0.090	0.184	-0.492	0.623
Sex_horseFilly (female under 3 years)	-0.098	0.115	-0.853	0.394
Sex_horseMare (female 3 years or over)	0.007	0.017	0.417	0.676
Sex_horseStallion (entire male 3 years or over)	-0.153	0.096	-1.597	0.111
Age_horse	-0.005	0.002	-3.031	0.002
ref= Crossbred Horse				
Analysis_breed Arabian	-0.023	0.054	-0.435	0.664
Analysis_breed Australian Stock Horse	-0.067	0.057	-1.192	0.233
Analysis_breed Standardbred	-0.122	0.057	-2.160	0.031
Analysis_breed Thoroughbred	-0.008	0.025	-0.311	0.756
Analysis_breedGaited	-0.097	0.068	-1.418	0.156
Analysis_breedHeavyHorse	-0.108	0.052	-2.096	0.036
Analysis_breedIberian	-0.173	0.061	-2.845	0.005
Analysis_breedNative	-0.103	0.144	-0.715	0.475
Analysis_breedOther	0.025	0.039	0.628	0.530
Analysis_breedPonyGroup	-0.145	0.057	-2.556	0.011
Analysis_breedWarmblood	-0.116	0.034	-3.385	0.001
Analysis_breedWestern_QuarterHorse	-0.102	0.034	-3.003	0.003
Ref= Pleasure Riding				
Analysis_DisciplineAdult riding club	-0.008	0.050	-0.162	0.872
Analysis_DisciplineBreeding_conformation	0.200	0.161	1.243	0.214
Analysis_DisciplineCompanion horse	0.049	0.071	0.690	0.490
Analysis_DisciplineCompetitive_riding	0.004	0.050	0.086	0.931
Analysis_DisciplineDressage	0.016	0.028	0.565	0.572
Analysis_DisciplineEndurance	0.129	0.074	1.756	0.079
Analysis_DisciplineEquitation	-0.066	0.078	-0.853	0.394
Analysis_DisciplineEventing	0.021	0.034	0.627	0.531
Analysis_DisciplineLiberty	-0.306	0.101	-3.019	0.003
Analysis_DisciplineMounted_games	0.030	0.071	0.425	0.671
Analysis_DisciplineOther	-0.007	0.047	-0.152	0.879
Analysis_DisciplinePony Club	0.046	0.049	0.934	0.351
Analysis_DisciplineRacing	0.197	0.109	1.809	0.071
Analysis_DisciplineShow-jumping	0.098	0.036	2.755	0.006
Analysis_DisciplineTherapy_horse	-0.183	0.116	-1.583	0.114
Analysis_DisciplineTrail riding/hacking	0.011	0.034	0.327	0.744
Analysis_DisciplineWestern_events	-0.074	0.051	-1.446	0.148
Analysis_DisciplineWestern_games	-0.020	0.085	-0.230	0.818
Analysis_DisciplineWorking Equitation	0.085	0.083	1.030	0.303
Analysis_DisciplineWorking_horse	0.060	0.078	0.770	0.441
ref= No professional saddle fitting				
Saddle_FitThis horse does not (yet) wear a saddle	-0.386	0.087	-4.425	0.000

Saddle_FitYes	-0.023	0.018	-1.290	0.197
ref= Bay				
Analysis_colourBlack	-0.025	0.032	-0.784	0.433
Analysis_colourBrown	-0.099	0.031	-3.165	0.002
Analysis_colourChesnut	-0.039	0.024	-1.600	0.110
Analysis_colourDilution	-0.080	0.042	-1.931	0.054
Analysis_colourGrey	-0.061	0.029	-2.074	0.038
Analysis_colourLeopard	-0.049	0.072	-0.687	0.492
Analysis_colourPalamino	-0.077	0.061	-1.249	0.212
Analysis_colourRoaned	-0.040	0.079	-0.509	0.611
Analysis_colourWhite	-0.033	0.202	-0.162	0.872
Analysis_colourWhitepatterned	-0.053	0.034	-1.571	0.116
ref= Handled Horses All my life				
ExperienceI've ridden/handled horses most of my life	0.066	0.022	3.022	0.003
ExperienceRider/handler with more than 8 years' experience	0.064	0.027	2.402	0.016
ExperienceRider/handler with up to 8 years' experience	0.175	0.040	4.418	0.000
ExperienceRider/handler with up to 5 years' experience	0.155	0.039	3.949	0.000
ExperienceRider/handler with up to 2 years' experience	0.098	0.063	1.557	0.120
ExperienceUp to 1 year's experience	0.152	0.090	1.681	0.093
ExperienceNo experience with horses	0.182	0.323	0.563	0.574
Ref= Communal barn				
Q54Paddock either at night or day and stable at other times	0.093	0.185	0.501	0.616
Q54Paddock or field 24/7 (with access to shelter)	0.062	0.184	0.335	0.738
Q54Stabled 24/7 and taken out for riding, lunging or walking in hand	-0.022	0.201	-0.108	0.914
Q54Stabled 24/7 with free 'play' time and ridden/ground work	0.082	0.194	0.424	0.672
Q54Stabled 24/7 with time each day to 'play' in an open area	0.081	0.199	0.409	0.683
Q54Usually paddocked but occasionally stabled (weather related)	0.085	0.185	0.458	0.647
Q54Usually stabled but occasionally paddocked	0.175	0.191	0.918	0.359
Ref= Beginner Rider				
Self_EvalA non-rider/non-horse person	-0.911	0.366	-2.489	0.013
Self_EvalA novice rider/horse handler	-0.127	0.082	-1.554	0.120
Self_EvalAn intermediate rider/horse handler	-0.169	0.083	-2.052	0.040
Self_EvalAn advanced rider/horse handler	-0.204	0.084	-2.436	0.015
Self_EvalAn elite rider	-0.420	0.110	-3.812	0.000

Partial Residual plot for Deceleration

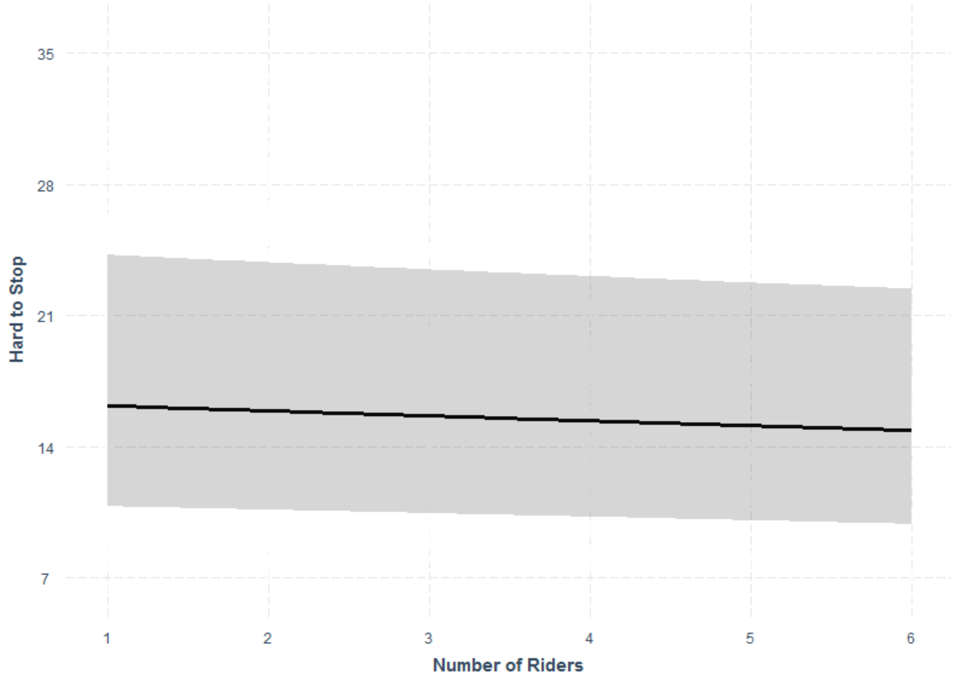

<u>Responsiveness Index</u>

```
modFinal <- lm(-1*log(18-mydata$SNS) ~ N_riders2+
                        Sex_horse:Age_horse +
                        Gender+
                        Analysis_Country+
                        Age+
                        Sex_horse+
                        Age_horse+
                        Analysis_breed+
                        Analysis_Discipline+
                        Experience+
                        Self_Eval, data = mydata)
```

Model Diagnostics

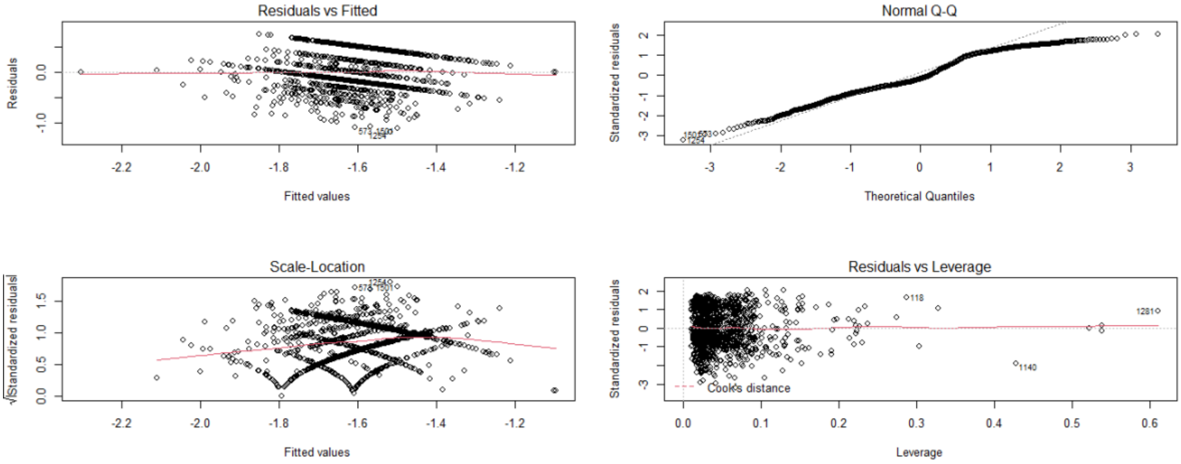

As expected, this is looking a little less like a normal distribution. It's not AWFUL though…

We'll now check the variance inflation factors (again, we have to take the interaction term out)

	GVIF	Df GVI	F^(1/(2*Df))	Square of GVIF^(1/(2*Df))
N_riders2	1.127185	1	1.061689	1.127184
Gender	1.264882	1	1.12467	1.264883
Analysis_Country	3.728391	10	1.068012	1.14065
Age	2.435211	7	1.065638	1.135584
Sex_horse	1.400553	4	1.043008	1.087866
Age_horse	1.128561	1	1.062337	1.12856
Analysis_breed	4.324708	12	1.062914	1.129786
Analysis_Discipline	7.937498	20	1.053155	1.109135
Experience	3.041691	7	1.0827	1.172239
Self_Eval	3.239048	5	1.124713	1.264979

No sign of an issue.

And Results….

<u>Model fit results</u>

Residual standard error: 0.381 on 1369 degrees of freedom
(158 observations deleted due to missingness)
Multiple R-squared: 0.09083, Adjusted R-squared: 0.04302
F-statistic: 1.9 on 72 and 1369 DF, p-value: 1.398e-05

(Somewhat lower R^2 here than the other two…)

Analysis of Variance type 3 table

	Sum Sq	Df	F value	Pr(>F)
(Intercept)	42.581	1	293.3271	< 2.2e-16
N_riders2	0.156	1	1.0758	0.300
Gender	0.178	1	1.2261	0.268
Analysis_Country	3.307	10	2.2782	0.012
Age	2.163	7	2.1291	0.038
Sex_horse	1.228	4	2.1147	0.077
Age_horse	1.862	1	12.8274	0.000
Analysis_breed	1.853	12	1.0637	0.387
Analysis_Discipline	2.53	20	0.8713	0.625
Experience	1.374	7	1.3526	0.222
Self_Eval	2.649	5	3.6494	0.003
Sex_horse:Age_horse	1.042	4	1.795	0.127
Residuals	198.729	1369		

Regression Coefficients

	Estimate	Std. Error	t value	Pr(>|t|)
(Intercept)	-2.017	0.118	-17.127	0.000
N_riders2	0.010	0.010	1.037	0.300
Ref= Female				
GenderMale	-0.066	0.060	-1.107	0.268
Ref= Australia				
Analysis_CountryBelgium	0.195	0.095	2.050	0.041
Analysis_CountryCanada	-0.012	0.039	-0.295	0.768
Analysis_CountryItaly	-0.064	0.107	-0.596	0.552
Analysis_CountryMexico	0.086	0.095	0.909	0.364
Analysis_CountryNew Zealand	-0.072	0.037	-1.942	0.052
Analysis_CountryOther	0.013	0.046	0.279	0.780
Analysis_CountrySouth Africa	-0.292	0.089	-3.280	0.001
Analysis_CountrySweden	0.030	0.089	0.342	0.732
Analysis_CountryUnited Kingdom of Great Britain and Northern Ireland	-0.041	0.037	-1.097	0.273
Analysis_CountryUnited States of America	0.023	0.034	0.665	0.506
Ref= 18-24				
Age25-34 years old	0.026	0.035	0.764	0.445

Age35-44 years old	0.022	0.037	0.589	0.556
Age45-54 years old	0.090	0.033	2.728	0.006
Age55-64 years old	0.023	0.036	0.644	0.520
Age65-74 years old	0.091	0.052	1.747	0.081
Age75 years or older	0.653	0.277	2.359	0.018
AgeUnder 18 - please complete under the supervision of a parent/guardian	0.054	0.055	0.988	0.323
Ref=Gelding				
Sex_horseColt (entire male under 3 years)	-0.170	0.364	-0.467	0.641
Sex_horseFilly (female under 3 years)	3.029	1.736	1.745	0.081
Sex_horseMare (female 3 years or over)	0.110	0.051	2.164	0.031
Sex_horseStallion (entire male 3 years or over)	0.196	0.216	0.905	0.366
Age_horse	0.009	0.002	3.582	0.000
Ref= Crossbred				
Analysis_breed Arabian	0.008	0.067	0.123	0.902
Analysis_breed Australian Stock Horse	0.077	0.071	1.081	0.280
Analysis_breed Standardbred	0.023	0.070	0.327	0.744
Analysis_breed Thoroughbred	-0.028	0.030	-0.938	0.349
Analysis_breedGaited	-0.054	0.082	-0.653	0.514
Analysis_breedHeavyHorse	0.156	0.063	2.484	0.013
Analysis_breedIberian	-0.068	0.076	-0.886	0.376
Analysis_breedNative	-0.172	0.179	-0.964	0.335
Analysis_breedOther	-0.034	0.048	-0.704	0.482
Analysis_breedPonyGroup	0.050	0.069	0.721	0.471
Analysis_breedWarmblood	-0.010	0.043	-0.229	0.819
Analysis_breedWestern_QuarterHorse	0.019	0.041	0.457	0.648
Ref= Pleasure Riding				
Analysis_DisciplineAdult riding club	0.025	0.062	0.413	0.680
Analysis_DisciplineBreeding_conformation	0.091	0.202	0.448	0.654
Analysis_DisciplineCompanion horse	-0.090	0.085	-1.054	0.292
Analysis_DisciplineCompetitive_riding	0.002	0.062	0.034	0.973
Analysis_DisciplineDressage	0.052	0.034	1.541	0.124
Analysis_DisciplineEndurance	0.182	0.091	1.992	0.047
Analysis_DisciplineEquitation	-0.005	0.094	-0.050	0.960
Analysis_DisciplineEventing	0.076	0.042	1.821	0.069
Analysis_DisciplineLiberty	0.217	0.132	1.653	0.098
Analysis_DisciplineMounted_games	-0.002	0.090	-0.025	0.980
Analysis_DisciplineOther	0.019	0.058	0.326	0.744

Analysis_DisciplinePony Club	0.056	0.059	0.935	0.350
Analysis_DisciplineRacing	0.015	0.129	0.119	0.905
Analysis_DisciplineShow-jumping	-0.005	0.044	-0.110	0.913
Analysis_DisciplineTherapy_horse	-0.049	0.141	-0.347	0.729
Analysis_DisciplineTrail riding/hacking	0.035	0.042	0.839	0.402
Analysis_DisciplineWestern_events	0.025	0.065	0.391	0.696
Analysis_DisciplineWestern_games	-0.056	0.103	-0.548	0.583
Analysis_DisciplineWorking Equitation	-0.088	0.100	-0.883	0.378
Analysis_DisciplineWorking_horse	0.130	0.093	1.400	0.162
Ref= Ridden all my life				
Ridden most of my life	-0.024	0.027	-0.885	0.376
ExperienceRider/handler with more than 8 years' experience	-0.024	0.033	-0.742	0.458
ExperienceRider/handler with up to 8 years' experience	-0.140	0.050	-2.812	0.005
ExperienceRider/handler with up to 5 years' experience	-0.014	0.048	-0.286	0.775
ExperienceRider/handler with up to 2 years' experience	-0.044	0.078	-0.566	0.571
ExperienceUp to 1 year's experience	0.060	0.112	0.541	0.588
ExperienceNo experience with horses	-0.254	0.401	-0.633	0.527
Ref= Beginner Rider				
Self_EvalA non-rider/non-horse person	0.049	0.457	0.107	0.914
Self_EvalA novice rider/horse handler	0.184	0.107	1.712	0.087
Self_EvalAn intermediate rider/horse handler	0.219	0.108	2.025	0.043
Self_EvalAn advanced rider/horse handler	0.300	0.109	2.739	0.006
Self_EvalAn elite rider	0.379	0.139	2.728	0.006
Interaction effects				
Sex_horseColt (entire male under 3 years):Age_horse	-0.008	0.059	-0.135	0.892
Sex_horseFilly (female under 3 years):Age_horse	-1.452	0.833	-1.743	0.082
Sex_horseMare (female 3 years or over):Age_horse	-0.008	0.004	-2.038	0.042
Sex_horseStallion (entire male 3 years or over):Age_horse	-0.003	0.015	-0.221	0.825
